# A peptide interfering with the dimerization of oncogenic KITENIN protein and its stability suppresses colorectal tumour progression

**DOI:** 10.1002/ctm2.871

**Published:** 2022-07-19

**Authors:** Sung Jin Kim, Eun Gene Sun, Jeong A Bae, Sehoon Park, Chang‐Soo Hong, Zee‐Yong Park, Hangun Kim, Kyung Keun Kim

**Affiliations:** ^1^ Department of Pharmacology Chonnam National University Medical School Gwangju Republic of Korea; ^2^ College of Pharmacy Sunchon National University Suncheon Republic of Korea; ^3^ School of Life Sciences Gwangju Institute of Science and Technology Gwangju Republic of Korea

**Keywords:** colorectal cancer, dimerization, KITENIN, Myo10, peptide cancer therapeutic

## Abstract

The stability of a protein, as well as its function and versatility, can be enhanced through oligomerization. KITENIN (KAI1 C‐terminal interacting tetraspanin) is known to promote the malignant progression of colorectal cancer (CRC). How KITENIN maintains its structural integrity and stability are largely unknown, however. Here we investigated the mechanisms regulating the stability of KITENIN with the aim of developing therapeutics blocking its oncogenic functions. We found that KITENIN formed a homo‐oligomeric complex and that the intracellular C‐terminal domain (KITENIN‐CTD) was needed for this oligomerization. Expression of the KITENIN‐CTD alone interfered with the formation of the KITENIN homodimer, and the amino acid sequence from 463 to 471 within the KITENIN‐CTD was the most effective. This sequence coupled with a cell‐penetrating peptide was named a KITENIN dimerization‐interfering peptide (KDIP). We next studied the mechanisms by which KDIP affected the stability of KITENIN. The KITENIN‐interacting protein myosin‐X (Myo10), which has oncogenic activity in several cancers, functioned as an effector to stabilize the KITENIN homodimer in the *cis* formation. Treatment with KDIP resulted in the disintegration of the homodimer via downregulation of Myo10, which led to increased binding of RACK1 to the exposed RACK1‐interacting motif (463–471 aa), and subsequent autophagy‐dependent degradation of KITENIN and reduced CRC cell invasion. Intravenous injection of KDIP significantly reduced the tumour burden in a syngeneic mouse tumour model and colorectal liver metastasis in an intrasplenic hepatic metastasis model. Collectively, our present results provide a new cancer therapeutic peptide for blocking colorectal liver metastasis, which acts by inducing the downregulation of Myo10 and specifically targeting the stability of the oncogenic KITENIN protein.

## Graphical abstract

In this study, we disclose a peptide tool for specifically blocking oncogenic KITENIN in CRC patients with higher KITENIN expression. The graphic working model shows the effects of the specific peptide, KDIP, on KITENIN stability. Myo10 regulates the oncogenic activity of KITENIN by stabilizing its dimerization. After binding to the C‐terminal region of KITENIN, KDIP interferes with KITENIN dimerization and induces the downregulation of Myo10, resulting the degradation of KITENIN.

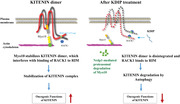

## 1 INTRODUCTION

Protein oligomerization can be defined as the arrangement of monomeric units of protein into homo‐ or hetero‐oligomers. Oligomerization creates protein configurations that allow for specificity and diversity of cellular functions, such as mediating gene expression and regulating the activity of enzymes, ion channels and receptors.^1^ Oligomerization is modulated by ligands, temperature and other proteins in the case of both homo‐ and hetero‐oligomers and provides the sites for allosteric regulation.^2^ Proteins form large assemblies through oligomerization without an increase in genome size, which may enhance protein stability.^3^, ^4^, ^5^ Therefore, many membrane‐associated and soluble proteins form homo‐oligomeric complexes in cells.^5^, ^6^ Homo‐oligomers exist most often as dimers and tetramers, which are about four times as common as hetero‐oligomers.^7^ Exposure of the hydrophobic interface of dimer proteins is known to cause conformational changes that can lead to a destabilization and degradation of protein via the proteasome or autophagy pathways.^8^, ^9^, ^10^ Thus, the modulation of oligomerization is an extremely promising therapeutic strategy for the treatment of diseases comprising oligomeric proteins,^11^ and a better understanding of the molecular aspects of protein oligomerization is needed.^12^

Colorectal cancer (CRC) is the third most common malignant cancer, and its metastasis is a major cause of cancer‐related death, in which the liver is most often involved.^13^ The use of chemotherapy and molecular targeted therapy to treat CRC has increased overall survival.^14^ However, the clinical benefits of these therapies are often short‐lived and restricted to a subpopulation of patients because of the development of distant metastasis and acquired resistances to targeted therapies.^15^, ^16^

Vangl proteins have an important function in neural tube formation during embryo development, and mutations in the *Vangl1* and *Vangl2* genes cause the neural tube defect craniorachischisis.^17^ We previously found that Vangl1, a membrane‐associated atypical tetraspanin with a long intracellular C‐terminal domain (CTD) (that we renamed KITENIN [KAI1 C‐terminal interacting tetraspanin]), binds to the C‐terminus of KAI1 and acts as a metastasis‐enhancing protein in CRC.^18^ CT‐26 mouse colon cancer cells overexpressing KITENIN show increased invasiveness and tumourigenicity and early hepatic metastasis resulting from KITENIN gain‐of‐function (KITENIN‐GOF). The functional KITENIN complex acts as an executor in regard to cell motility and thereby controls CRC cell invasion, which contributes to promoting metastasis.^19^ Furthermore, KITENIN levels are positively correlated with advanced stage^19^ and lymph node metastasis^20^ in CRC. The presence of an unconventional EGFR‐independent signal of EGF, the KITENIN/ErbB4‐Dvl2‐c‐Jun axis, also mediates increased CRC cell invasiveness and represents poor responses to cetuximab.^21^, ^22^ The KITENIN axis also plays an important role in colorectal carcinogenesis within an *adenomatous polyposis coli*‐loss‐associated environment.^23^ Thus, these reports suggest that the KITENIN axis is a molecular target for developing therapeutics to block the malignant progression of CRC. However, apart from analyses of expression and post‐translational modification with regard to the biochemical characteristics of KITENIN, no study has been done of how KITENIN stability is regulated and which molecules are closely involved in this regulation.

In this study, we investigated the biochemical characteristics regulating the stability of KITENIN. We theorized that by identifying the biochemical features maintaining the structural integrity of KITENIN, we could then target KITENIN to develop new therapeutics for CRC patients expressing higher KITENIN levels. Here, we report the homodimerization of KITENIN through the intracellular CTD as one of the biochemical features contributing to maintaining the stability of KITENIN. We also report that a KITENIN dimerization‐interfering peptide (KDIP) derived from the CTD interferes with the formation of the KITENIN homodimer and thereby degrades the KITENIN protein in an autophagy‐dependent manner via an increased binding of KITENIN with RACK1. We found that the 463–471 aa‐deleted KITENIN construct formed a homodimer but did not bind to RACK1 and was not degraded after KDIP treatment, indicating that this sequence in the CTD works as a RACK1‐interacting motif (RIM). To find molecules that interact with KITENIN and mediate the effects of KDIP, we performed immunoprecipitation analysis and detected the KITENIN‐interacting protein, myosin‐X or Myosin10 (Myo10), which functioned to stabilize the KITENIN homodimer in the *cis* formation. After treatment with KDIP, the downregulation of Myo10 was induced via proteasomal degradation. In in vivo mouse tumour models with the higher levels of KITENIN expression, KDIP significantly reduced the tumour burden and suppressed colorectal liver metastasis. Furthermore, a positive correlation was found between the expression of *KITENIN* and that of *Myo10* in colorectal adenocarcinoma of The Cancer Genome Atlas (TCGA). The present results therefore provide a tool for specifically blocking the oncogenic actions of KITENIN in CRC patients with higher KITENIN expression.

## 2 MATERIALS AND METHODS

### 2.1 Cell culture and reagents

Cell lines were purchased from the Korean Cell Line Bank (KCLB, Seoul, Republic of Korea) and were routinely screened for mycoplasma contamination. CT‐26‐WT‐iRFP‐Neo cells were purchased from Imanis Life Sciences (Rochester). Cells were cultured in RPMI‐1640 medium or DMEM containing 10% foetal bovine serum (GenDEPOT), 100 units/ml of penicillin, and 100 μg/ml of streptomycin (Corning) at 37°C in a humidified atmosphere containing 5% CO_2_. Cells were passaged before reaching confluence. 3‐MA, Brefeldin A, chloroquine, cycloheximide, MG132 and rapamycin (Sigma) were treated at the indicated concentrations. Composition of several cell lysis buffers for the preparation of whole‐cell lysate is as follows; native lysis buffer: 150‐mM sodium chloride, .1% Triton X‐100, 50‐mM Tris pH 8.0, 1‐M EDTA; regular lysis buffer: 150‐mM sodium chloride, 1% Triton X‐100, .1% SDS, 50‐mM Tris pH 8.0, 2‐M EDTA, added protease and phosphatase inhibitors; IP lysis buffer: 25‐mM Tris–HCl, pH 7.4, 150‐mM NaCl, 1‐mM EDTA, 1% NP‐40, 5% glycerol.

### 2.2 Plasmids and siRNA

Expression constructs were generated by PCR‐based methods: V5‐tagged, Myc‐tagged, HA‐tagged KITENIN, GST‐tagged deletion mutants of KITENIN, Δ463–471‐KITENIN, and His‐tagged KITENIN. All constructs were confirmed by sequencing. pEGFP‐N1‐RACK1 was a gift from Anna Huttenlocher (Addgene plasmid #41088). All siRNAs used for gene silencing were obtained from Santa Cruz Biotechnology. Each consisted of a mixture of several sequences, thus eliminating sequence‐specific diversity.

### 2.3 Acrylamide gel staining and PMF analysis

Coomassie brilliant blue G 250 staining was performed for the visualization of proteins separated by SDS–PAGE. Briefly, the gels were fixed for 30 min in fixation solution (30% ethanol, 2% phosphoric acid in water) on a shaking platform. After the fixed gels were washed with washing solution (2% phosphoric acid in water), they were equilibrated for 30 min in equilibrium solution (18% ethanol, 15% ammonium sulphate and 2% phosphoric acid in water). Protein bands visualized through staining were punched out for in‐gel digestion followed by MALDI‐TOF mass spectrometry. All digested peptides were verified by peptide mass fingerprinting (PMF) analysis (Genomine, Korea).

### 2.4 Antibodies and immunoprecipitation

Antibodies against the following proteins were obtained from the indicated suppliers: Na^+^/K^+^ ATPase, GFP and tubulin (Santa Cruz Biotechnology); c‐Jun, p‐c‐Jun, ERK, p‐ERK, c‐MET, p‐c‐MET and GST (Cell Signaling Technology); V5, Myc (MBL); HA and β‐actin (Sigma); KITENIN (Atlas); His and RACK1, LC3, p62, Myo10, and eukaryotic elongation factor 2 (eEF2) (Abcam). They were used with appropriate secondary antibodies (Thermo). For transient transfection analyses, Caco2, HCT116 and 293T cells were transfected with various plasmids and harvested for immunoblot analysis 48 h after transfection. For most assays using stable cell lines, mixed polyclonal cells were used to exclude clonal variation. Cellular proteins were separated, transferred and immunoblotted as previously described.^18^ Cell lysates from Caco2, HCT116 and 293T cells were used for immunoprecipitation experiments as previously described.^19^

### 2.5 Subcellular fractionation

Cytoplasmic and membrane fractions were prepared by a subcellular protein fractionation kit for cultured cells (Thermo Scientific) as previously described.^24^ Each fraction was resolved by SDS–PAGE and probed for KITENIN by tagging antibodies to V5 and Myc. Fraction purity was analysed by probing for tubulin for the cytoplasm and Na^+^–K^+^ ATPase for the membrane protein.

### 2.6 Cell invasion assay

Cell invasion was measured using the transwell migration apparatus as described.^18^ Briefly, cultured cells were seeded into the top of a 24‐well invasion chamber assay plate (Costar). Conditioned DMEM medium containing 10 μg/ml of fibronectin (Calbiochem) and 1% FBS was added to the bottom chamber as a chemoattractant. After 16 h (Caco2) or 48 h (HCT116) of incubation, the cells were stained. Cells at the top surface of the filters were wiped off with a cotton ball, and migrated cells on the bottom surface were counted in four random squares of .5 mm × .5 mm on each filter. The results are shown as the mean ± SEM of the number of cells per field for at least three independent experiments.

### 2.7 In situ proximity ligation assay

Control‐Caco2/empty vector (EV) and Caco2/KITENIN‐Myc/HA cells were embedded in 1% agarose and fixed overnight with 10% neutral buffered formalin solution. After fixation, the cells were stained at 4°C overnight with primary antibodies against a tagging of KITENIN. Then secondary antibodies against mouse and rabbit antibodies (Duolink in situ proximity ligation assay Probes, Sigma‐Aldrich) were added. According to the manufacturer's protocol, the brightfield detection system (Duolink Brightfield in situ detection reagents, Sigma‐Aldrich, Darmstadt, Germany) was applied to the slides (Duolink proximity ligation assay reagents Brightfield Protocol). The density of the glomerular signals was analysed by using confocal microscopy.

### 2.8 Pull‐down assay

GST‐KITENIN or its derivatives were immobilized on glutathione‐sepharose beads. Myc‐tagged KITENIN was incubated with GST‐tagged KITENIN or its derivative proteins immobilized on glutathione‐sepharose as indicated. The bound protein complexes were eluted and analysed by SDS–PAGE followed by IB. To detect in vitro KITENIN homodimerization, streptavidin‐bound GST‐KITENIN was incubated with Ni‐NTA‐purified KITENIN‐His and KDIP (5 μM) in the pull‐down buffer (50‐mM Tris.Cl [pH 7.5], 1‐mM dithiothreitol [DTT] 4% [v/v] glycerol, .1 mg/ml of BSA, 5‐mM MgCl_2_, 1‐mM ATP and 50‐mM NaCl) at 4°C for 2 h. Following incubation, the beads were washed thoroughly using washing buffer (pull‐down buffer with 300‐mM NaCl) and bead bound proteins were resolved on SDS–PAGE followed by immunoblot analysis using indicated antibodies.

### 2.9 RT‐PCR and Q‐PCR

RNA preparation and reverse transcription were performed as previously described.^18^ The RT‐PCR exponential phase was set for 30 cycles to allow for quantitative comparison of the various cDNAs developed from identical reactions on a GeneAmp PCR system (Eppendorf). Real‐time PCR was carried out using a Rotor‐gene (Qiagen). Reactions were run in triplicate in three independent experiments. Expression data were normalized to the geometric mean of the housekeeping gene GAPDH to control for variability in expression levels and were analysed as previously described.^23^

### 2.10 Autophagosome staining

To test whether KDIP induces autophagy in KITENIN‐expressing Caco2 cells, cells were grown on coverslips for 24 h. Cells were washed with PBS and then stained with Cyto‐ID green fluorescence reagents (Enzo Life Sciences, Plymouth Meeting, PA) for 1 h at 37°C in a cell culture incubator. Cells were washed with PBS and mounted with VECTASHIELD mounting medium containing nuclear DAPI (Vector Lab). Cells were imaged by confocal microscope.

### 2.11 Grating‐coupled interferometry (GCI)

Grating‐coupled interferometry (GCI) experiments were performed with the Creoptix WAVE delta (Creoptix AG). The 4PCP WAVE chip (thin quasi‐planar polycarboxylate surface, Creoptix) was conditioned with borate buffer (.1‐M borate pH 9.0, 1‐M NaCl, Creoptix) and .2× PBS‐P+ (Cytiva). The anti‐GST antibody (MBL, polyclonal) was immobilized on the chip surface using standard amine coupling; activation with 1:1 mix of 400‐mM *N*‐(3‐dimethylaminopropyl)‐*N*′‐ethylcarbodiimide hydrochloride (Cytiva) and 100‐mM *N*‐hydroxysuccinimide (NHS, Cytiva) for 7 min at 10 μl/min, antibody immobilization with anti‐GST antibody (10 μg/ml) in 10‐mM sodium acetate pH 5.0 (Cytiva) for 7 min at 10 μl/min, passivation with .05 % (w/v) BSA in 10‐mM sodium acetate pH 5.0 for 7 min at 10 μl/min, followed by 1‐M ethanolamine pH 8.5 (Cytiva) for 7 min at 10 μl/min. Respective GST‐tagged ligands (recombinant GST‐alone, GST‐tagged WT‐KITENIN and GST‐tagged KITENIN‐CTD) were captured until the desired density was reached. Multi‐cycle kinetic analyses for protein peptide interaction were performed at 25°C with a 1:2 dilution series from 125 nM for KDIP (Peptron) with 1× PBS‐P+ running buffer (Cytiva). Various concentrations of KDIP were injected over the chip surface at 30 μl/min with an association time of 60 s followed by a dissociation time of 60 s. The obtained results were analysed using the Creoptix WAVE control software. One‐to‐one binding models with bulk correction were used for the experiments.

### 2.12 In vivo tumour growth and hepatic metastases model

All animal experiments were performed under the guidelines of the Chonnam National University Medical School Research Institutional Animal Care Committee, and all experimental protocols were approved by the committee (CNU IACUC‐H‐2019‐3).

The CT‐26 cell/syngeneic mouse model was used to investigate the in vivo effects of KDIP on colorectal tumourigenesis, as it is reported that a syngeneic mouse tumour model is good for testing the anticancer effects of candidate substances.^25^, ^26^ Male Balb/c mice (5‐week old) were purchased from DaMul Science, Korea and acclimated for 1 week prior to subcutaneous injection of syngeneic CT‐26/EV (3 × 10^6^) or CT‐26/KITENIN cells (1 × 10^6^) into the dorsum. Tumour volume was calculated using the following equation: 𝑉 = 1/2 × 𝑎 × 𝑏 × 2, where 𝑎 and 𝑏 are the longest and shortest diameters of the tumour (in millimetres), respectively. Tumour volume was measured every other day for 14 days to verify the effects of KDIP. All mice were sacrificed after Day 30 (Figure 7B), and the subcutaneous tumour grafts were surgically excised and weighed.

For hepatic metastasis analysis, 5‐ to 6‐week‐old Balb/c mice were obtained from OrientBio, Inc. (Seongnam, Korea) and housed in metal cages with free access to water and food. A syngeneic mouse model of colorectal liver metastasis was established by an infusion of tumour cells into the portal system via intrasplenic injection.^27^ In brief, CT‐26/KITENIN‐iRFP‐expressing cells (2 × 10^5^ cells/mouse) were injected into the spleen. Splenectomy was performed 15 min after an injection of CT‐26 cells. Mice were randomly assigned to two groups: scrambled peptide and KDIP (1, 5 mg/kg). Each peptide was given via intravenous injection six times for 2 weeks after 7 days of inoculation of tumour cells. Metastatic tumour nodules in the liver with a diameter of >1.0 mm were counted using a microscope, and a metastasis score was allocated based on nodule size as follows: 0 (no gross metastasis), 1 (tumour size >1 mm), 2 (tumour size 1 >5 mm) and 3 (tumour size >10 mm). The metastasis score was obtained by multiplying the number and the score of nodules. Fluorescence images of liver nodules expressing iRFP were taken using a Fluorescence‐labelled Organism Bioimaging Instrument (Cellgentek, Korea) and total fluorescence emitted from liver nodules was measured and compared between groups.

### 2.13 Recovery, matrix effect of KDIP extraction and stability of KDIP in mice serum

Recovery and matrix effect for serum extraction were calculated by peak area of KDIP using three types of samples (unextracted sample, extracted sample, post‐extracted spiked sample). Recovery was calculated using extracted sample and post‐extracted spiked sample, by following equation: recovery (%) = 100 × peak area of extracted sample/post‐extracted spiked sample. Matrix effect was determined using post‐extracted spiked sample and unextracted sample, by following equation: matrix effect (%) = 100 × ([peak area of post‐extracted spike sample/peak area of unextracted sample]‐1). A volume of 10‐μl serum was used and KDIP 100 pmol was spiked to each sample. Each type of sample was triplicated and 2.5 pmol of KDIP from each sample was analysed by LC–MS/MS as described.^28^

Experiment for stability in serum was conducted with seven different incubation time (0, 5, 10, 20, 40, 80, 160 min). KDIP 10 pmol was spiked to 10 μl of serum, and samples were incubated at room temperature. Samples for each incubation time were triplicated.

### 2.14 Tissue processing and sample preparation for LC–MS/MS analysis

At 20 min after fifth intravenous injection of KDIP, tumour and liver tissues were collected, immediately frozen using liquid nitrogen and ground with a pestle and mortar. One part of resultant powder by weight was homogenized with two parts of lysis buffer containing 8‐M urea, 25‐mM Tris–HCl (pH 8.5) by volume and was placed in Eppendorf tubes. This lysate was sonicated three times for 2 s with 2‐s intervals of rest at 20% amplitude on ice. Insoluble debris was removed by obtaining supernatant after centrifugation at 14 000 *g*, 4°C for 15 min.

Peptide extraction from serum or tissue lysate was performed by solid‐phase extraction using Oasis HLB Cartridge 1‐cm^3^ (30 mg). HLB cartridges were conditioned using ACN (1 ml) and 50% ACN, .5% FA (1 ml), followed by equilibration using .5% FA (2 ml). A volume of 10‐μl serum and 20 μl of tissue lysate were added by .5% FA to be 1 ml and were loaded on equilibrated cartridges. Cartridges were washed with .5% FA (3 ml), and then peptide was eluted by 50% ACN, .5% FA (500 μl). Eluates were vacuum‐dried using Speed‐Vac and were stored at −80°C until use. Dried samples were reconstituted in .5% FA 50 and 5 μl of sample was loaded on column for LC–MS/MS analysis.

### 2.15 LC–MS/MS analysis

Each sample was analysed using an Agilent 1100 Series high‐performance liquid chromatography pump (Agilent Technologies, USA) coupled to an LTQ linear ion trap mass spectrometer (Thermo Finnigan, USA). Separation of peptide was carried out on a homemade C18‐fused silica column (100‐μm internal diameter) with nano‐electrospray ionization interface. Split flow configuration was used for an adjustment of column flow rate to ∼250 nl/ml. Mobile phase A (.5% FA) and B (.5% FA in ACN) were used for 30‐min gradient. It was started with a 20‐min gradient for 0%–30% solvent B, and then solvent B was ramped to 80% in 1 min. Overall, 80% solvent B was maintained for 4 min, followed by a 1‐min gradient to 0%, and solvent B was held at 0% for 4 min. Peptide was quantified by multiple reaction monitoring (MRM) in positive ion mode, with a spray voltage of 2.3 kV. Following transitions were chosen for KDIP: 501.1 → 437.5 (b_17_^+5^), 501.1 → 477.55 (b_19_^+5^), 501.1 → 546.5 (b_17_^+4^), 501.1 → 592.25 (b_19_ + H_2_O^+4^), 501.1 → 596.5 (b_19_^+4^). Furthermore, truncated forms (ARVRRRGPRRWRLVSDEAV, VRRRGPRRWRLVSDEAV) of KDIP that were detected during stability test were also analysed by following transitions: 468.5 → 404.8 (b_16_^+5^), 468.5 → 444.85 (b_18_^+5^), 468.5 → 505.7 (b_16_^+4^), 468.5 → 551.35 (b_18_ + H_2_O^+4^), 468.5 → 555.7 (b_18_^+4^), 528.45 → 448.85 (b_14_^+4^), 528.45 → 499.0 (b_16_^+4^), 528.45 → 598.15 (b_14_^+3^), 528.45 → 659.25 (b_16_ + H_2_O^+3^) and 528.45 → 664.95 (b_16_^+3^).

### 2.16 Analysis of TCGA datasheet

The TCGA datasheet of colorectal adenocarcinoma with information about tumour stage and mRNA expression levels (PanCancer Atlas, Colorectal Cancer project) were retrieved from cBioProtal (http://www.cbioportal.org/). The correlations between the expression levels of *KITENIN* and *Myo10* were estimated using Pearson correlation analysis and linear regression analysis. A correlation coefficient *r* greater than 0 indicates a positive association between the expressions of two genes.

### 2.17 Statistics

Experimental differences were tested for statistical significance by using ANOVA followed by Tukey HSD post hoc test or Student's *t* test. All statistical tests were two‐sided, and *p* values of less than .05 were assigned to statistical significance. Statistical analyses were performed using the PASW Statistics 20 software (SPSS, an IBM Company, Chicago, IL).

## 3 RESULTS

### 3.1 The intracellular C‐terminal region of KITENIN contributes to homodimerization and stability of the KITENIN protein

The KITENIN complex plays an important role in the progression and metastasis of CRC,^19^, ^21^, ^23^ but little is known about how the protein maintains its structural integrity and stability. Given that homo‐oligomerization of molecules allows proteins to form large, stable structures,^1^, ^3^, ^4^, ^5^ we first examined whether the KITENIN protein forms a homo‐oligomeric complex.

**FIGURE 1:**
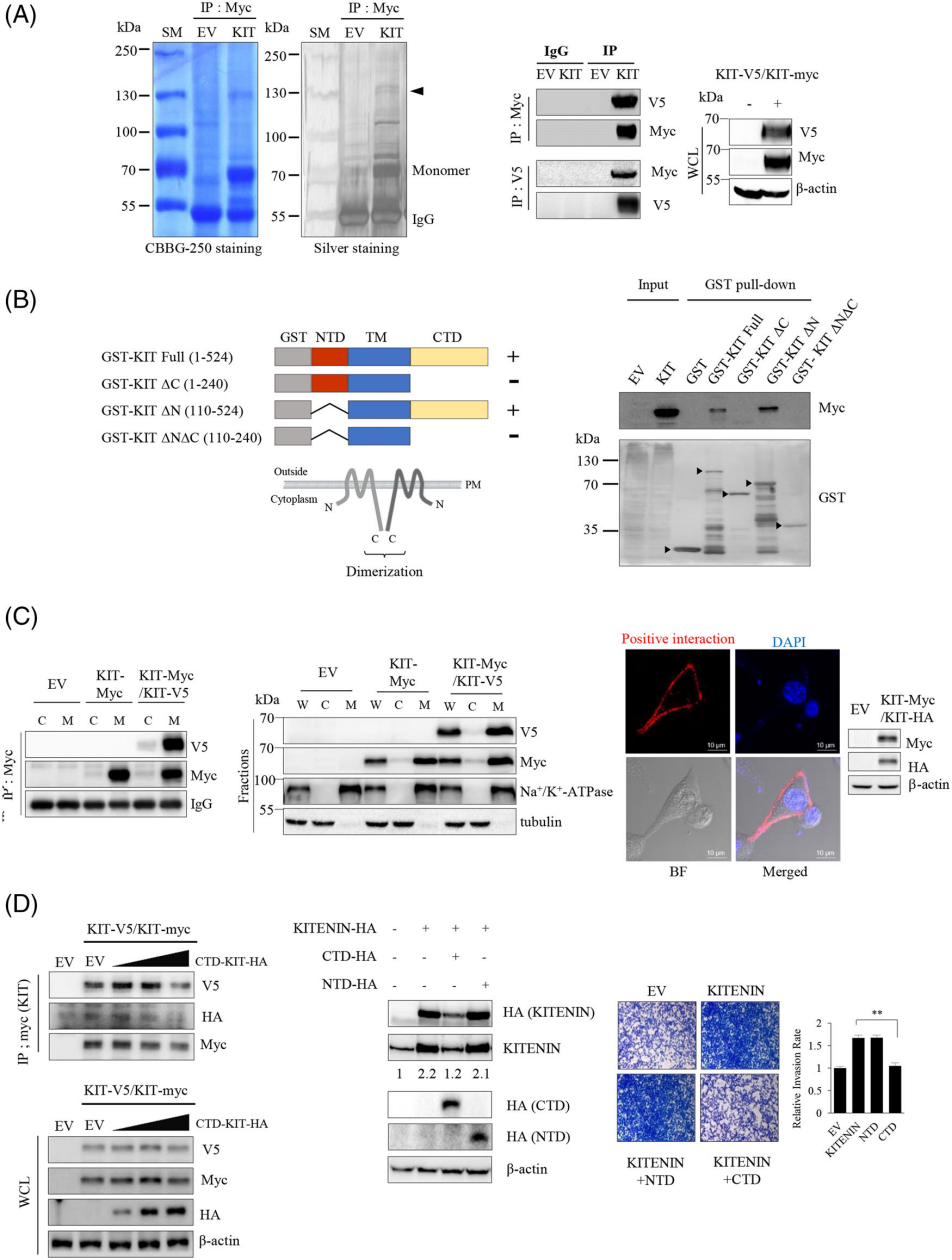
Homodimerization of KAI1 C‐terminal interacting tetraspanin (KITENIN) through its C‐terminal domain (CTD). (A) KITENIN‐Myc and KITENIN‐V5 form homodimers. HEK293T cells were transfected with plasmid encoding KITENIN‐myc. Purified Myc‐tagged KITENIN‐specific eluates were prepared, followed by SDS–PAGE and staining with Coomassie blue (CBBG‐250) or silver (left panel). The arrow head on the gel indicates the protein identified by PMF (peptide mass fingerprinting) analysis, which showed that KITENIN‐Myc interacted with the endogenous KITENIN and formed a homodimer. Immunoprecipitation assay was performed in Caco2 cells co‐transfected with KITENIN‐Myc or ‐V5 (middle panel). Expressions of KITENIN‐Myc or ‐V5 in whole‐cell lysate (WCL) were shown (right panel). (B) Schematic diagram of GST‐tagging domain‐deleted KITENIN mutants: full‐length, ΔC (1–240), ΔN (110–524), ΔNΔC (110–240) (left panel). Each mutant was expressed in *Escherichia coli* as a GST fusion protein, immobilized on glutathione‐sepharose beads and analysed on an SDS–PAGE gel, which showed the comparative size of the GST fusion proteins (black arrowheads, right panel). Overall 10% of the lysate used in the pull‐down was run for input. (C) Homodimerization of KITENIN occurs at the cell membrane. Caco2 cells were transfected with empty vector (EV), KITENIN‐Myc and KITENIN‐V5 for 48 h and subjected to subcellular fractionation into cytosol and membrane fractions. Tubulin and Na^+^/K^+^‐ATPase were used as cytosolic and membrane markers, respectively. Immunoprecipitation assay was performed using cytosol and membrane fractions (left panel). Expressions of KITENIN‐Myc or ‐V5 in WCL, cytosol (C) and membrane (M) fractions were shown (middle panel). Subcellular distribution of KITENIN homodimer detected by in situ proximity ligation assay (right panel). Caco2 cells transfected with KITENIN‐Myc and KITENIN‐HA were grown on cover slips. Red fluorescent dots indicate the KITENIN homodimer. (D) Expression of KITENIN‐CTD reduces the dimerization of KITENIN. Caco2 cells co‐expressing KITENIN‐V5/Myc were transfected with increasing doses of KITENIN‐CTD‐HA. After 48 h of incubation, the cells were immunoprecipitated with anti‐Myc antibody and detected by the indicated antibodies (left panel). Caco2 cells were transfected with KITENIN, KITENIN and NTD (1–240), and KITENIN and CTD (110–524) for 48 h and subjected to immunoblot assay (middle panel) and in vitro transwell invasion assay (right panel). Numerals indicated a quantification of the KITENIN bands normalized to the corresponding β‐actin bands (middle panel). The pictures shown represent three independent experiments (right panel). The histogram represents invading cells, which were counted at the four chosen areas and shown as bar graphs (mean ± SEM, *n*  =  3).

To identify proteins that interact with KITENIN, we first immunoprecipitated the total protein content of KITENIN‐myc‐overexpressing 293T cells with Myc‐Tag antibody and separated the precipitated proteins by size using SDS–PAGE. A 130‐kDa band was isolated and identified as KITENIN by PMF (Figure 1A, left). As the band size was approximately double that of the KITENIN monomer, we assumed that it was a homodimerized form of KITENIN. We then performed Western blot and co‐immunoprecipitation (co‐IP) assays in cells transfected with two different KITENIN tagging constructs. Immunoblots using antibody against the V5 or myc epitope, respectively, confirmed the co‐expression of monomers of KITENIN in transfected cells (Figure 1A, right). We observed an interaction between KITENIN‐V5 and KITENIN‐myc in HEK293T cells (Figure 1A, middle), but also in CRC cell lines, such as Caco2 and HCT116 cells (Figure S1A). To show the presence of endogenous KITENIN dimer in non‐transfected CRC cells, we chose the HCT116 cells, which express the highest endogenous KITENIN levels,^21^ and performed Western blot assay using a regular SDS–PAGE gel in the reducing/denaturing condition. The KITENIN homodimer was preserved in HCT116 and Caco2 cells under the reducing/denaturing condition (Figure S1B).

To identify the domains participating in the dimeric association, each domain of the KITENIN protein was expressed using a GST bacterial expression system. The KITENIN protein was divided into four forms: full‐length, deleted CTD (ΔC; NTD), deleted N‐terminal domain (ΔN; CTD) and deleted N‐ and CTDs (ΔNΔC; TM, transmembrane domain) (Figure 1B, left). Lysates of 293T cells expressing full‐length KITENIN‐myc and purified GST‐fused truncated forms were pulled down with glutathione‐sepharose beads and then incubated with myc antibody to detect the site of dimerization in KITENIN. When the KITENIN C‐terminal region was deleted, the protein was not detected by myc antibody, indicating that the dimerization of KITENIN occurred through the intracellular CTD (KITENIN‐CTD, Figure 1B, right). To determine where in the cell KITENIN is dimerized, we performed IP of the cell membrane fraction. We observed that KITENIN, a cell‐membrane‐spanning protein, forms a dimer in the cell membrane (Figure 1C, left). The dimerization spot could also be observed as red fluorescence by confocal microscopy using the proximity ligation assay (Figure 1C, right). To determine whether this dimerization of KITENIN occurred in the *cis* (at the surface of the same cell) formation (Figure S1C), co‐IP experiments were performed. The Caco2 cells were transfected with either KITENIN‐V5 or KITENIN‐myc or co‐transfected with KITENIN‐V5 and KITENIN‐myc. The reciprocal interactions between KITENIN‐myc and KITENIN‐V5 were observed in co‐transfected Caco2 cells (Figure S1D). Thus, KITENIN homodimers exist exclusively in the *cis* form.

Next, because we showed that dimerization occurs through the intracellular C‐terminal region, we examined whether expression of KITENIN‐CTD (244–524 aa) affected the dimerization of KITENIN. Interestingly, the dimerization of KITENIN was decreased after ectopic overexpression of KITENIN‐CTD (Figure 1D, left). Also, after co‐expression of KITENIN‐CTD, the increased KITENIN level (Figure 1D, middle) or cell invasion (Figure 1D, right) by co‐transfection of wild‐type KITENIN was suppressed to a level, similar to that with expression of EV. By contrast, the increased KITENIN level and cell invasion due to co‐transfection of wild‐type KITENIN were not affected by co‐expression of the N‐terminal region (NTD) of KITENIN (Figure 1D, middle and right). These results suggested that ectopic expression of KITENIN‐CTD interferes with KITENIN homodimerization at the protein level. Overall, these results imply that KITENIN homodimers are crucial for the stability and oncogenic function of KITENIN protein and that the suppression of KITENIN‐GOF by KITENIN‐CTD might be via a dysregulation of homodimerization. That is, KITENIN‐CTD may downregulate homodimerization by competitive interaction with other full‐length KITENIN molecules but also by disrupting the homodimers via a modulation of unidentified molecules responsible for maintaining the stability of the KITENIN dimer. However, the finding of no specific interaction between KITENIN‐CTD‐HA and KITENIN‐MYC (Figure 1D, left) seems to rule out the former possibility.

### 3.2 Identification of candidate peptide sequences within KITENIN‐CTD that interfere with the dimerization of KITENIN

Because an overexpression of KITENIN‐CTD inhibited the dimerization of KITENIN, we next tried to identify the responsible region within the CTD by use of serial deletion constructs. The forced expression of three deletion constructs (1–339, 1–394, 1–449) resulted in increased cell invasion, compared with that of wild‐type KITENIN, whereas the 1–487 construct resulted in decreased cell invasion, which is similar to that of KITENIN‐CTD. These results indicated that the 449–487 region of KITENIN might be responsible for the inhibitory effect on dimerization and cell invasion (Figure S2A,B). To narrow down the region, we designed several peptides by dividing the 449–487 region of KITENIN into 10‐mers or less and fused these to a cell‐permeable peptide sequence (CPP) derived from the human transcription factor Hph‐1 (Figure S2C).^29^, ^30^ We tested the inhibitory effect of each peptide on KITENIN dimerization. As shown in Figure S2D, the 463–471 peptide sequence was the most effective for inhibiting the dimerization and cell invasion caused by KITENIN overexpression. This peptide sequence was thus named KDIP. To find the most appropriate peptide‐linker for the effect of KDIP on efficient cell permeability, dimer interference and suppression of invasion, various other CPPs or tumour‐homing sequences were attached to the KDIP (Figure S3A) and tested. These results showed that the dimerization of KITENIN was inhibited by KDIP‐CPPs or tumour‐homing peptides to a similar degree, but CPP (Hph‐1) showed the best inhibitory effect on cell invasion (*p* < .001) (Figure S3B,C). Thus, the KDIP peptide derived from KITENIN‐CTD could effectively inhibit the formation of dimers and cell invasion caused by an overexpression of KITENIN.

### 3.3 KDIP decreases homodimerization and stability of the KITENIN protein

**FIGURE 2:**
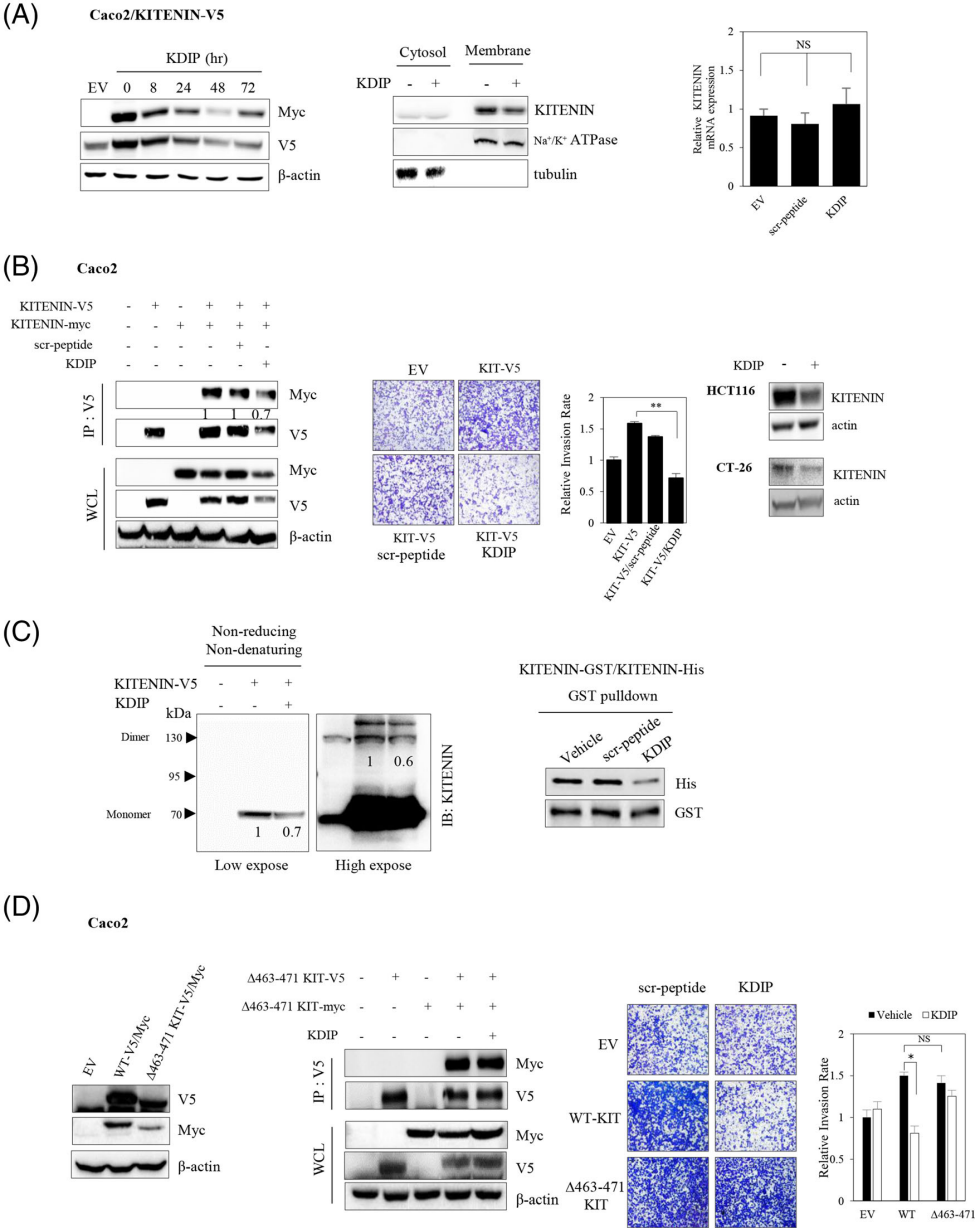
KAI1 C‐terminal interacting tetraspanin (KITENIN) dimerization‐interfering peptide (KDIP) suppresses KITENIN‐induced enhanced cell invasion through the inhibition of the homodimerization of KITENIN. (A) KDIP decreases the amount of KITENIN protein, but not the expression of *KITENIN*. Caco2/KITENIN‐V5 cells were transfected with KITENIN‐myc. After 24 h, cells were treated with KDIP (1 μM) for the indicated times (left panel) and subjected to subcellular fractionation into cytosol and membrane fractions (middle panel). Relative *KITENIN* expression in scrambled peptide (scr‐pep)‐ or KDIP‐treated Caco2 cells (right panel). After 48 h of treatment, KITENIN transcripts in cells were analysed by the 2^−ΔΔ^ *^CT^* method using Q‐PCR. Data represent mean values ± SEM (*n*  =  3). (B) KDIP directly inhibits the formation of KITENIN homodimers and the upregulated cell invasion by overexpressed KITENIN but also decreases endogenous KITENIN level. Caco2 cells were co‐transfected with KITENIN‐V5/Myc and treated with or without KDIP or scrambled peptide (scr) and were immunoprecipitated with anti‐V5 antibody and detected by indicated antibodies. Numerals indicated a quantification of the MYC bands normalized to the corresponding V5 bands (left panel). Caco2 cells were transfected with empty vector or KITENIN‐V5 and treated with scrambled peptide or KDIP and subjected to in vitro transwell invasion assay (middle panel). The histogram represents invading cells, which were counted at four chosen areas and shown by bar graphs (mean ± SEM, *n* = 4, ^**^ *p* < .01). HCT116 and CT‐26 cells were treated with or without KDIP (1 μM) for 12 h and analysed by immunoblotting with an anti‐KITENIN antibody (right panel). (C) Formation of both the KITENIN monomer and dimer is reduced by treatment with KDIP. Caco2 cells were transfected with KITENIN‐V5 and treated with or without KDIP. Cell lysates were run on a native SDS–PAGE gel in the non‐reducing/non‐denaturing condition and immunoblotted with an anti‐KITENIN antibody (left panel). Protein size is indicated by an arrowhead. Intensities of the 130‐kDa KITENIN homodimer band and the 70‐kDa KITENIN monomer band were compared in low or high expose state. Numerals indicated a quantification of the bands observed (left panel). KITENIN‐GST and KITENIN‐His were expressed in bacteria, in vitro purified, and mixed with scr‐peptide or KDIP. After GST pull‐down, KITENIN‐His attached to KITENIN‐GST in glutathione‐sepharose beads was detected by anti‐His antibody (right panel). (D) Degradation of KITENIN after KDIP requires the intact KDIP sequence (463–471 peptide in Figure S3C, RACK1‐binding site) within KITENIN. Caco2 cells were transfected with KITENIN‐V5/Myc or Δ463–471 KIT‐V5/Myc and treated with KDIP for 24 h, and immunoreactive V5‐tagging and Myc‐tagging proteins were examined after immunoprecipitation with anti‐V5 antibody (left panel). Influence of forced expression of Δ463–471 KITENIN on the effect of KDIP on cell invasion (right panel). After 24 h of KDIP treatment, cells were subjected to invasion assay. The pictures and histogram of the invasion assay were obtained as in Figure 1D (Mean ± SEM, *n* = 4). Asterisk (^*^ *p* < .05) indicates a significant difference between groups. NS, no significant difference between groups

We next checked whether the suppressive effects of KITENIN‐CTD overexpression on the level of KITENIN and its dimerization (Figure 1D) were at the transcription or protein level. We found that levels of KITENIN transcripts were roughly the same after forced expression of KITENIN‐CTD as after forced expression of KITENIN‐WT or KITENIN‐NTD (Figure S3D), and that KITENIN transcript was not affected after treatment with KDIP, compared with those of scr‐peptide treated or no treatment group (Figure 2A, right). These data indicated that the reduction of the protein amount by KITENIN‐CTD or KDIP could be considered effects on the protein level. As with KITENIN‐CTD (Figure 1D), treatment with KDIP decreased KITENIN in the cell membrane fraction, with the greatest reduction at 48 h (Figure 2A, left and middle). Also, as for KITENIN‐CTD, treatment with KDIP reduced the homodimerization of KITENIN (Figure 2B, left) and cellular invasion (Figure 2B, middle) by overexpressed KITENIN. Moreover, treatment with KDIP also decreased endogenous KITENIN protein levels in non‐transfected HCT116 and CT‐26 cells (Figure 2B, right). We rechecked the effect of KDIP on the dimerization of KITENIN by SDS–PAGE in the non‐reducing/non‐denaturing condition. We found that the 130‐kDa KITENIN homodimer band and the 70‐kDa KITENIN monomer band were also decreased after treatment with KDIP (Figure 2C, left). In addition, the dimerization of KITENIN protein was directly reduced by treatment with KDIP when the two purified KITENIN proteins with different tags were pulled down in vitro (Figure 2C, right). Thus, we supposed that the reduction of the KITENIN dimer after treatment with KDIP may be the result of degradation of KITENIN after exposure of specific regions important to protein stability due to dissociation of the KITENIN dimer by KDIP. Dimerization of the KITENIN protein may prevent the exposure of these regions via structural hindrance.

To next check whether the 463–471 peptide sequence is necessary for the increased KITENIN degradation after KDIP treatment, we made a mutant KITENIN construct (Δ463–471‐KITENIN, Figure 2D, left). The Δ463–471‐KITENIN formed a homodimer like WT‐KITENIN, but the effects of KDIP on reducing dimerization and KITENIN protein were not observed (Figure 2D, middle). In addition, the overexpression of Δ463–471‐KITENIN increased cell invasion as with the overexpression of WT‐KITENIN, but the inhibitory effect of KDIP on increased cell invasion by Δ463–471‐KITENIN was not observed (Figure 2D, right). Thus, the 463–471 peptide sequence is required for the subsequent degradation of KITENIN after treatment with KDIP and degradation of the KITENIN dimer after KDIP results in an inhibition of the increased cell invasion caused by overexpressed KITENIN.

Next, we performed GCI to investigate the binding of KDIP with the WT‐KITENIN or KITENIN‐CTD, which is essential for KITENIN homodimerization. These experiments confirmed that KDIP binds to the KITENIN‐CTD (*K*_d_ = .897 μM) as well as to WT‐KITENIN (*K*_d_ = 2.722 μM) (Figure S4). We speculate that the slightly higher *K*_d_ value of KDIP bound to WT‐KITENIN is possibly due to the folding of whole protein, in contrast with KITENIN‐CTD, which contains the intracellular C‐terminal region alone.

### 3.4 Treatment with KDIP accelerates binding of RACK1 with KITENIN and the degradation of KITENIN

**FIGURE 3:**
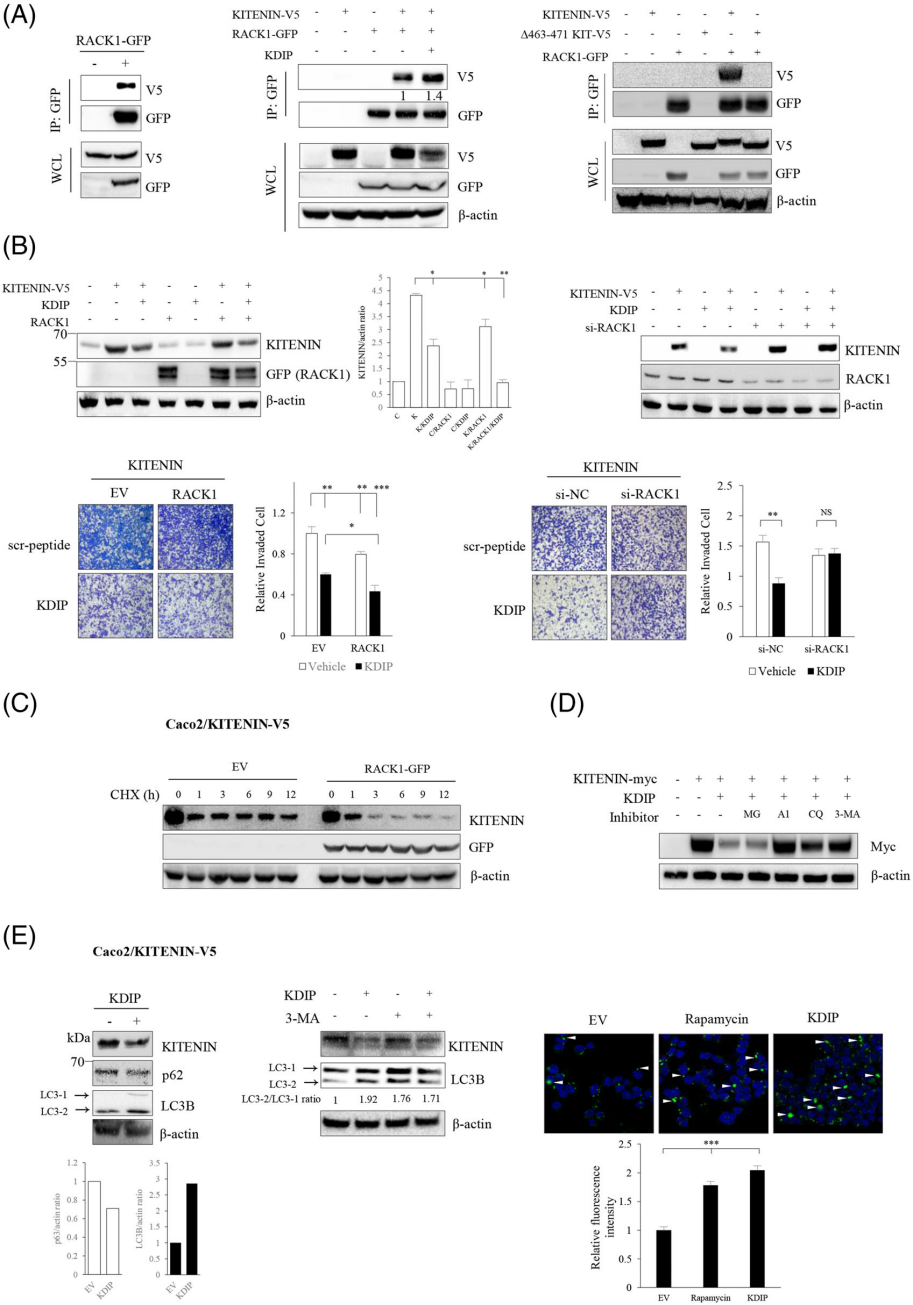
RACK1 binds to KAI1 C‐terminal interacting tetraspanin (KITENIN) and KITENIN dimerization‐interfering peptide (KDIP) stimulates the degradation of KITENIN by inducing increased binding of RACK1 to KITENIN. (A) RACK1 specifically binds to KITENIN. Caco2/KITENIN‐V5 cells were transfected with RACK1‐GFP for 48 h, immunoprecipitated with anti‐GFP antibody and immunoblotted with anti‐V5 antibody to detect KITENIN bound with RACK1 (left panel). Caco2 cells were transfected with KITENIN‐V5 and/or RACK‐GFP. After 24 h, cells were treated with KDIP (1 μM) for 24 h and immunoprecipitated with anti‐GFP antibody (middle panel). Caco2 cells were transfected with KITENIN‐V5, or Δ463–471‐KITENIN‐V5 and/or RACK1‐GFP, for 48 h and immunoprecipitated with anti‐GFP antibody to detect interaction between Δ463–471 KITENIN and RACK1 (right panel). The proteins in whole‐cell lysate (WCL) were detected with the indicated antibody. (B) Changes in the effect of KDIP on degradation of KITENIN according to the expression status of RACK1. Caco2 cells were transfected with KITENIN‐V5 and/or RACK1‐GFP (left) or KITENIN‐V5 and/or si‐RACK1 (right), and their expression after treatment with KDIP (1 μM, 24 h) was detected with the indicated antibody (upper) or subjected to invasion assay (lower). The pictures and histogram of the invasion assay were obtained as in Figure 2D. (C) Changes in the amount of KITENIN protein according to RACK1 expression. Caco2/KITENIN‐V5 cells were transfected with empty vector or RACK1‐GFP for 48 h, treated with cycloheximide (CHX, 20 ng/ml) for the time points indicated (hours), and examined by immunoblot analyses. (D) Degradation of KITENIN after KDIP treatment shows a lysosome‐autophagy pathway‐dependent pattern. Caco2 cells transfected with KITENIN‐myc for 48 h were treated with KDIP (1 μM) for 24 h. During this experimental period, cells were treated again with MG132 (10 μM) and A1 (Bafilomycin A1, 1 μM), CQ (chloroquine, 100 μM), or 3‐MA (1 mM) for 4 and 12 h, respectively, before the cells were harvested. The amount of KITENIN protein was checked by an anti‐myc antibody. (E) KDIP stimulates autophagic degradation of KITEINN. Caco2/KITENIN‐V5 cells were treated with KDIP for 24 h and assessed by immunoblot analyses using an anti‐KITENIN, an anti‐p62, or an anti‐LC3B antibody (left panel). LC3B and p62 were used as a marker of autophagy. Caco2/KITENIN‐V5 cells were treated with KDIP and/or 3‐MA for 24 h and changes in the expression of LC3B were examined by immunoblot analyses (middle panel). Autophagic vacuoles (indicated by white arrow heads) were stained with Cyto‐ID green dye following treatment with Rapamycin or KDIP (5 μM) in Caco2/KITENIN‐V5 cells (right panel). The histogram of the autophagy assay was represented (mean ± SEM, *n* = 3) and asterisk (^***^ *p* < .001) indicates a significant difference between groups.

KITENIN (Vangl1) is a membrane protein, and its localization at the membrane is essential for its role in maintaining polarity as a component of the planar cell polarity (PCP) pathway.^31^, ^32^ RACK1 is known to maintain PCP signalling and the membrane localization of Vangl2.^33^ We previously found that RACK1 serves as an adaptor protein for the molecules involved in the downstream signalling of the KITENIN complex in which RACK1 might control the level of Dvl2 and KITENIN via autophagy‐dependent degradation.^27^ To check whether RACK1 directly interacts with KITENIN, we performed an IP experiment using antibody against GFP in Caco2 cells co‐expressing RACK1‐GFP and KITENIN‐V5 and found that RACK1 bound with KITENIN (Figure 3A, left). Also, RACK1 directly interacted with endogenous KITENIN in non‐transfected HCT116 cells (Figure S5A). Interestingly, treatment with KDIP after the overexpression of RACK1‐GFP caused an increased binding of KITENIN to RACK1 and a profound reduction of KITENIN protein in Caco2 cells co‐expressing RACK1‐GFP and KITENIN‐V5 (Figure 3A, middle). Moreover, when compared with those of non‐transfected cells, a further reduction of endogenous KITENIN protein was found after treatment with KDIP in RACK1‐GFP transfected HCT116 and CT‐26 cells (Figure S5B). It can be inferred that KITENIN dimerization was broken after treatment with KDIP, which caused conformational changes that exposed the masked area by dimerization and led to the increased binding of RACK1 to KITENIN. We hypothesized that the 463–471 aa sequence of KITENIN was the responsible masked area, and we tested this by co‐IP using Δ463–471‐KITENIN‐V5 and GFP‐RACK1. In contrast with WT‐KITENIN, Δ463–471‐KITENIN did not bind with RACK1 in Caco2 cells (Figure 3A, right) and in HCT116 cells (Figure S5C). Collectively, these results indicated that the 463–471 peptide sequence within KITENIN is required for binding of RACK1 with KITENIN, and further that this site is hindered from binding with RACK1 when KITENIN is in a dimerized form. We thus named the 463–471 aa sequence in the CTD an RIM.

We next checked whether RACK1 is needed for the degradation of KITENIN by treatment with KDIP or transfection of KITENIN‐CTD. As shown, KITENIN was further reduced by KDIP (Figure 3B, left) or KITENIN‐CTD (Figure S6A, left) when RACK1 was overexpressed. Transfection of RACK1 alone did not change the KITENIN content; however, when cells were treated with KDIP or KITENIN‐CTD in the presence of RACK1 overexpression, the KITENIN content was further reduced. These results indicated that treatment with KDIP or transfection of KITENIN‐CTD induced KITENIN decomposition through an increased binding of KITENIN with RACK1. When the RACK1 was knocked down by treatment with siRNA specific to RACK1, the effect of KDIP (Figure 3B, right) or KITENIN‐CTD (Figure S6A, right) on reducing the amount of KITENIN protein was blunted. In contrast to the KITENIN content, increased cell invasion by KITENIN was suppressed by the expression of RACK1 (Figure 3B, left). When RACK1 was co‐overexpressed, KDIP showed a greater inhibitory effect on cell invasion than when KITENIN was expressed alone (Figure 3B, left), whereas under treatment with si‐RACK1, KDIP did not show an inhibitory effect on cell invasion (Figure 3B, right). Likewise, the reduced cell invasion by KITENIN‐CTD was further accelerated by the overexpression of RACK1 (Figure S6B, left), but treatment with si‐RACK1 blocked an inhibitory effect of KITENIN‐CTD on cell invasion (Figure S6B, right).

### 3.5 RACK1 facilitates KITENIN degradation after treatment with KDIP via the autophagy pathway

RACK1 is reported to bind to ribosomes and affect mRNA translation and protein synthesis.^34^ To exclude the possibility that RACK1 affected KITENIN protein synthesis, we pretreated the cells with cycloheximide, a translation blocker, and examined the KITENIN contents. We found that the degradation of KITENIN protein was accelerated by overexpressed RACK1 in the cycloheximide‐pretreated cells compared with EV‐transfected control cells (Figure 3C). For the most part, proteins are degraded through either the proteasome or the lysosome. To delineate which pathway is responsible for the degradation of KITENIN by KDIP or KITENIN‐CTD, we examined the KITENIN content in cells after pretreatment with the proteasome inhibitor MG132, the lysosome inhibitors bafilomycin A1 (BFA1) and chloroquine (CQ), or the autophagy inhibitor 3‐MA. The degradation of KITENIN by KDIP (Figure 3D) or KITENIN‐CTD (Figure S6C) was attenuated by BFA1, CQ, and 3‐MA but not MG132, suggesting that KDIP or KITENIN‐CTD promotes the degradation of KITENIN through the lysosome‐autophagy pathway. Moreover, the level of LC3, one of the features of autophagy, was increased after treatment with KDIP (Figure 3E, left) and this effect was diminished by treatment with the autophagy inhibitor 3‐MA (Figure 3E, middle). In addition, to analyse the effect of treatment with KDIP on induction of autophagy, we observed changes in the autophagosome after treatment with KDIP via cyto‐ID staining. The formation of the autophagosome was increased by KDIP, similar to the effect of rapamycin, a positive control (Figure 3E, right).

### 3.6 Myo10 is responsible for maintaining the stable KITENIN dimer state that hinders binding of RACK1 to RIM

**FIGURE 4:**
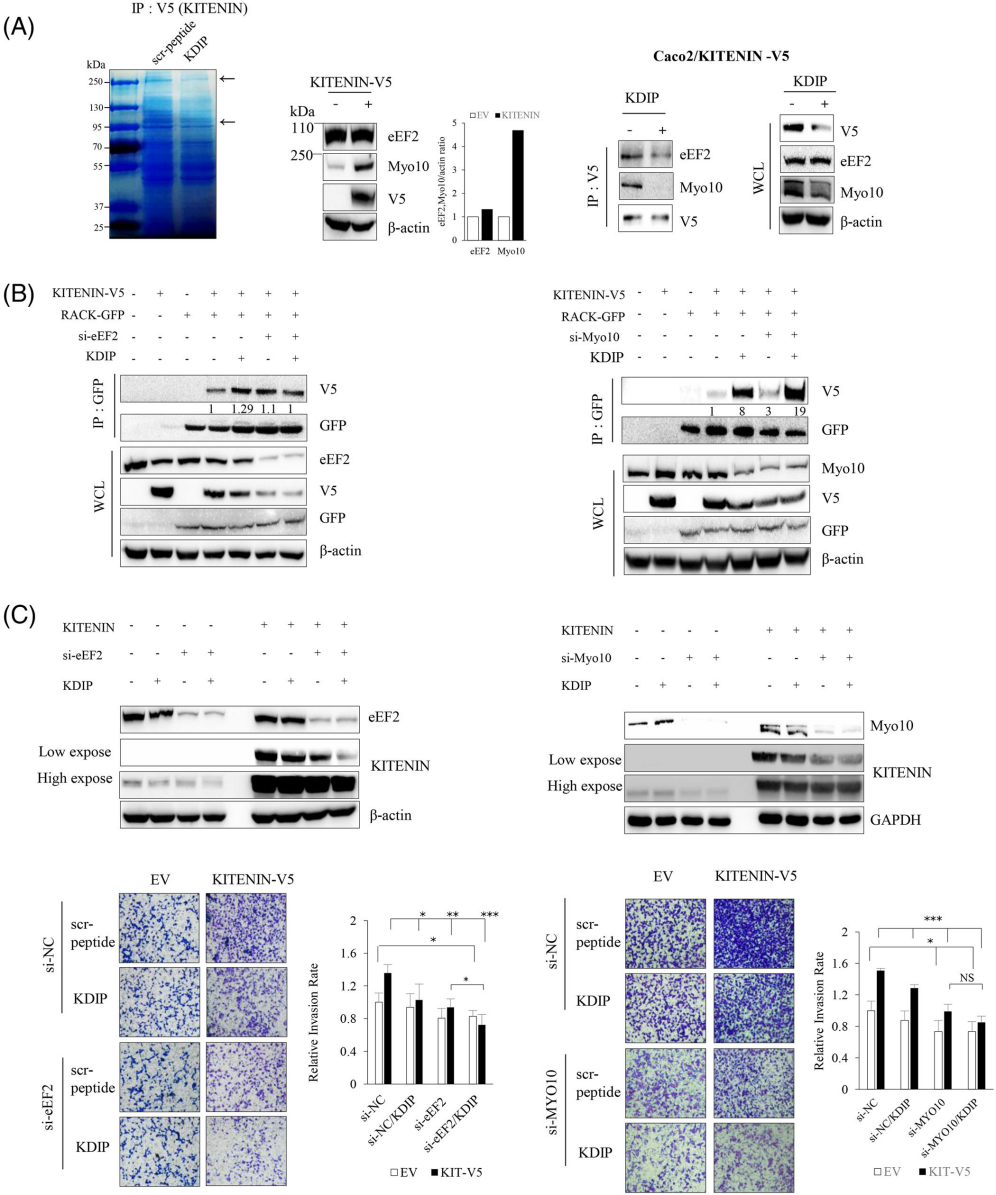
Identification of candidate molecules that mediate the effects of KAI1 C‐terminal interacting tetraspanin (KITENIN) dimerization‐interfering peptide (KDIP). (A) Identification of KITENIN‐binding proteins that showed a quantitatively changed pattern after treatment with KDIP. Caco2/KITENIN‐V5 cells were treated with scr‐peptide or KDIP, followed by immunoprecipitation with anti‐V5 antibody. The eluates were separated on an SDS–PAGE gel, stained with Coomassie blue and prepared for PMF (peptide mass fingerprint) analysis (left panel). Arrows indicate proteins that showed weakened binding to KITENIN after KDIP. Changes in the identified KITENIN‐binding proteins in KITENIN‐transfected cells (middle panel). The expression patterns of eukaryotic elongation factor 2 (eEF2) and Myosin‐X (Myo10) identified by PMF analysis were examined in Caco2/KITENIN‐V5 cells. Decreased expression pattern of KITENIN‐binding proteins after KDIP (right panel). Caco2/KITENIN‐V5 cells were treated with KDIP, followed by immunoprecipitation with anti‐V5 antibody and immunoblotted with the indicated antibodies. (B) Changes in the binding profile of KITENIN with RACK1 by the knockdown of the identified KITENIN‐binding proteins, eEF2 and Myo10. Caco2 cells were transfected with KITENIN‐V5 and/or RACK1‐GFP under knockdown of eEF2 (left panel) or Myo10 (right panel) via siRNA transfection, and treated with KDIP (1 μM) for 24 h. The cells were immunoprecipitated with anti‐GFP antibody (RACK1) and immunoblotted with anti‐V5 antibody (KITENIN). Numerals indicated a quantification of the V5 bands normalized to the corresponding GFP bands. (C) Myo10 mediates the inhibitory effects of KDIP on KITENIN level and cell invasion. Caco2 cells were transfected with empty vector or KITENIN‐V5 under knockdown of eEF2 (left panel) or Myo10 (right panel) via siRNA transfection and treated with KDIP (1 μM) for 24 h. The cells were analysed by immunoblot assay to detect the KITENIN level (upper panel) or invasion assay (lower panel). The pictures and histogram of the invasion assay were obtained as in Figure 2D (mean ± SEM, *n* = 4, ^*^ *p* < .05, ^**^ *p* < .01, ^***^ *p* < .001).

As we observed that increased binding of RACK1 with KITENIN after treatment with KDIP caused the degradation of KITENIN, we hypothesized that the interaction of RACK1 with KITENIN is hindered when KITENIN exists as a homodimer because the RACK1 interaction domain is structurally hidden or masked. If another KITENIN‐interacting molecule responsible for maintaining the stability of the KITENIN dimer is modulated after treatment with KDIP, it is possible that the KITENIN dimer will be disrupted after KDIP treatment, and that RIM will also be subsequently exposed and thereby KITENIN is degraded by interaction of RACK1 with RIM. To test this possibility, KDIP‐treated Caco2 cells were immunoprecipitated with KITENIN antibody and separated by SDS–PAGE, and the bands that changed after KDIP treatment were cut out for protein sequencing. In this way, we identified or Myo10 and eEF2 (Figure 4A, left), one of several actin‐based motor molecules in the myosin superfamily^35^ and a member of the GTP‐binding translation elongation factor family that is essential for protein synthesis,^36^ respectively. When we compared the endogenous levels of the two proteins between KITENIN‐transfected and parental Caco2 cells, the amount of Myo10 was increased in KITENIN‐transfected cells, whereas that of eEF2 was not (Figure 4A, middle). In addition, after treatment with KDIP in Caco2/KITENIN‐V5 cells, binding of KITENIN to eEF2 was a little decreased, but binding of KITENIN to Myo10 was actually abolished (Figure 4A, right). Moreover, treatment with KDIP caused a reduction in Myo10 levels (Figure 4A, right). To confirm whether both proteins may contribute to the stability of KITENIN, we examined the binding of RACK1 to KITENIN after knockdown of both proteins by treatment with siRNA specific for Myo10 or eEF2. Interestingly, the interaction of RACK1 with KITENIN was not substantially affected by the downregulation of eEF2 or Myo10 (Figure 4B). However, the binding of RACK1 to KITENIN was increased after treatment with KDIP and this effect of KDIP was augmented after the knockdown of Myo10 (Figure 4B, right), compared with the knockdown of eEF2 (Figure 4B, left). Consistently, KDIP further reduced the amount of KITENIN under eEF2‐knockdown (Figure 4C, left), whereas the further inhibitory effect of KDIP on the KITENIN level was not observed when Myo10 was knocked down (Figure 4C, right). To confirm the contribution of both proteins to the stability of KITENIN, we examined whether knockdown of Myo10 or eEF2 by siRNA changed the effect of KDIP on cell invasiveness of the KITENIN‐transfected cells. We found that the increased cell invasion caused by the overexpression of KITENIN was significantly reduced by the downregulation of these two proteins (Figure 4C). However, the inhibitory effect of KDIP on the increased cell invasiveness caused by overexpressed KITENIN was still obvious when eEF2 was knocked down (Figure 4C, left) but not under knockdown of Myo10 (Figure 4C, right).

### 3.7 Myo10 interacts with the transmembrane portion of KITENIN and stabilizes the KITENIN homodimer

Myo10 has a motor or head domain with a nucleotide‐binding site and an actin‐binding site; an IQ or neck domain, which binds three molecules of calmodulin; a C‐terminal tail domain that has a single α‐helix region followed by a coiled‐coil region presumably involved in dimerization; three PEST sequences, which confer sensitivity to certain proteases; three pleckstrin homology domains; a myosin tail homology 4 (MyTH4) domain, which binds to microtubules; and a band 4.1, ezrin, radixin, merlin (FERM) domain.^35^, ^37^ Myo10 localizes to the tips of filopodia, which are actin‐rich finger‐like protrusions found at the leading edge of cells,^37^ and is believed to be involved in cell migration, wound healing, adhesion to the extracellular matrix, guidance toward chemoattractants, neuronal growth‐cone path finding, embryonic development and tumourigenesis.^38^

**FIGURE 5:**
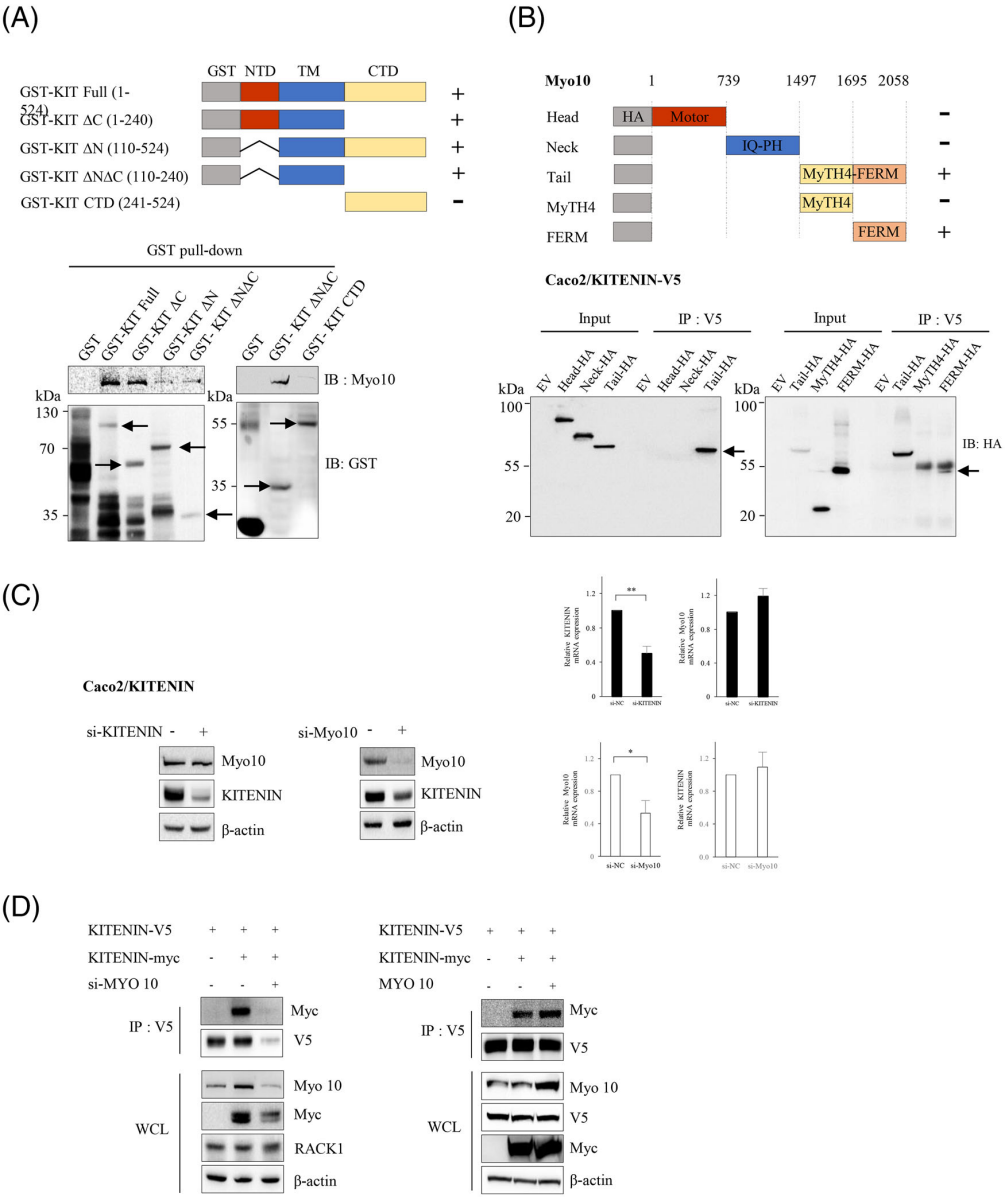
Myosin‐X (Myo10) interacts with the transmembrane portion of KAI1 C‐terminal interacting tetraspanin (KITENIN) and thereby stabilizes the KITENIN homodimer. (A) Myo10 binds to the transmembrane portion of KITENIN. Bacterially expressed proteins from GST‐tagged KITENIN deletion constructs were purified, incubated with cell lysates containing Myo10 and pulled down. The bound protein complexes were eluted and analysed by SDS–PAGE followed by immunoblotting. The interaction was examined by probing the blots with an anti‐Myo10 antibody. Positive bands were detected in KITENIN full‐length and the ΔC (KITENIN without the intracellular C‐terminal region), the ΔN (KITENIN without the intracellular N‐terminal region) and the ΔNΔC (KITENIN without the intracellular N‐terminal and C‐terminal regions, but with the transmembrane region) constructs, but not in the C‐terminal domain (CTD) (intracellular C‐terminal region without membrane portion) construct. Arrows point to the right size of each protein. (B) The C‐terminal FERM domain of Myo10 interacts with the transmembrane portion of KITENIN. Schematic representations of Myo10‐HA deletion mutants are shown. The open box indicates each functional domain of Myo10. Three (left panel) or two (right panel) deletion mutants of HA‐Myo10 were expressed in Caco2/KITENIN‐V5 cells, and cell lysates were immunoprecipitated with anti‐V5 antibody and immunoblotted with anti‐HA antibody to detect the interaction between KITENIN and HA‐tagging Myo10. Each Myo10 deletion mutant that bound KITENIN is indicated by an arrow. (C) Myo10 acts as a modulator of the expression of KITENIN. Caco2/KITENIN‐V5 cells were transfected with si‐KITENIN or si‐Myo10 for 48 h and analysed by immunoblot analyses with the indicated antibodies (left and middle panel) or by RT‐PCR analyses (right panel). The expression of Myo10 was not affected by knockdown of KITENIN (left panel), but the expression of KITENIN was nearly absent under knockdown of Myo10 (middle panel). The mRNA histograms showing a gene levels of KITENIN and Myo10 after knockdown of Myo10 and knockdown of KITENIN, respectively (right panel). The expression of *Myo10* or *KITENIN* was not affected by knockdown of KITENIN or Myo10, respectively. (D) Formation of the KITENIN dimer is nearly disrupted under the knockdown of Myo10. Caco2 cells co‐transfected with KITENIN‐V5 and/or KITENIN‐Myc were transfected with si‐Myo10 for 48 h and were immunoprecipitated with anti‐V5 antibody and analysed by the indicated antibodies (left panel). Caco2/KITENIN‐V5 cells were co‐transfected with KITENIN‐Myc and/or Myo10 for 48 h and were immunoprecipitated with anti‐V5 antibody and analysed by the indicated antibodies (right panel).

We hypothesized that the Myo10 level is modulated after treatment with KDIP and that this change is essential to the effect of KDIP on degradation of KITENIN. We thus asked whether the modulation of Myo10 affects the stability of KITENIN. First, we examined which domain of Myo10 interacts with what portion of KITENIN. Interestingly, Myo10 bound to the transmembrane portion of KITENIN (Figure 5A) via its C‐terminal FERM domain (Figure 5B). Because Myo10 binds with the underlying actin cytoskeleton via its N‐terminal head domain,^31^ this result indicated that Myo10 interacts with the intracellular cytoplasmic loop within the transmembrane portion of KITENIN. Because the FERM domain has been reported to have a role in attaching other proteins to the membrane,^33^ we supposed that the interaction of KITENIN with the C‐terminal FERM domain is also important to the stabilization of the KITENIN dimer on the plasma membrane.

Knockdown of KITENIN did not affect the level of Myo10, whereas knockdown of Myo10 resulted in reduced KITENIN levels in Caco2 cells (Figure 5C), and in HCT116 and CT‐26 cells (Figure S6D), indicating that Myo10 acts as a modulator of the protein levels, but not gene level of KITENIN. Moreover, the knockdown of Myo10 resulted in a reduced formation of KITENIN dimer (Figure 5D) but also enhanced the interaction of RACK1 with KITENIN and increased the degradation of KITENIN (Figure 4B, right). Therefore, the decomposition of the KITENIN homodimer, and the subsequent degradation of KITENIN, after treatment with KDIP is triggered by a downregulation of Myo10.

Considering that after treatment with KDIP, dimerization was not disrupted, nor was stability affected in KITENIN mutants in which the 463–471 region was deleted (Figure 2D), we hypothesized that the binding of KDIP, which is composed of a CPP (11 aa) and the RIM (463–471 aa, 9 aa), to the C‐terminal region of KITENIN creates an artificial and transient RACK1‐binding site. Furthermore, we supposed that KDIP requires the endogenous 463–471 region of KITENIN to interfere at least with KITENIN dimerization, but not degradation. We thus tested the effects of treatment with KDIP on deletion mutants of KITENIN with various intracellular C‐terminal regions (1–339, 1–394, 1–449 and 1–487 aa), including full‐length KITENIN (1–524 aa). We found that the 1–487 mutant and full‐length KITENIN were affected after KDIP treatment (Figure S7A). This indicated that the C‐terminal 449–487 region (39 aa) of KITENIN is necessary for KDIP to bind with KITENIN (Figure S7B) and that the endogenous RIM (463–471 aa) site in KITENIN is necessary for KDIP action after binding: triggering the physical dissociation of the KITENIN dimer in a wedge‐like action, which subsequently leads to degradation of KITENIN via autophagy after binding of RACK1 to the RIM.

### 3.8 Myo10 is decreased after KDIP via proteasomal degradation

Because the degradation of KITENIN after treatment with KDIP was somewhat recovered by pretreatment with MG132, whereas it was fully restored by pretreatment with the inhibitors of the lysosome‐autophagy pathway (Figure 3D), we further asked whether the proteasomal or lysosomal degradation pathway is responsible for the reduction of Myo10 by KDIP. For these experiments, we pretreated cells with the proteasome inhibitor MG132, the lysosome inhibitor CQ and the autophagy inhibitor 3‐MA. We found that the reduction in Myo10 after KDIP treatment was restored by pretreatment with MG132, but not CQ or 3‐MA (Figure 6A), and that the degradation of KITENIN facilitated by KDIP was somewhat attenuated by MG132. Because cells were pretreated with MG132 for 4 h before harvesting owing to cellular toxicity, these results suggest that the reduction in Myo10 after KDIP treatment is a critical event for the degradation of KITENIN by KDIP. Interestingly, Myo10 was also decreased after the transfection of KITENIN‐CTD (Figure 6B). To validate the effect of MG132 on the reduction of Myo10 after KDIP treatment, we performed an in vitro phenotype analysis using the cell invasion assay. In these experiments, pretreatment with the proteasome inhibitor MG132 affected neither the cell invasion nor the effect of KDIP in parent Caco2 cells but blocked the inhibitory effect of KDIP on the increased cell invasion in KITENIN‐overexpressed Caco2 cells (Figure 6C). These results indicated that KITENIN‐CTD or KDIP caused the disintegration of the KITENIN homodimer via a downregulation of Myo10.

FIGURE 6KAI1 C‐terminal interacting tetraspanin (KITENIN) dimerization‐interfering peptide (KDIP) decreases Myosin‐X (Myo10) via the E3‐ligase Nrdp1‐mediated proteasomal degradation. (A) Decreased Myo10 after KDIP is restored after pretreatment with MG132 but not lysosome inhibitor or autophagy inhibitor. Caco2 cells transfected with KITENIN‐V5 were treated with KDIP (1 μM) for 24 h. During this experimental period, cells were treated again with MG132 (10 μM) and chloroquine (100 μM) or 3‐MA (1 mM) for 4 and 12 h, respectively, before cells were harvested. Amounts of Myo10 and KITENIN proteins were checked by immunoblot analyses. (B) Decreased Myo10 after the transfection of KITENIN‐C‐terminal domain (CTD). Caco2 cells expressing empty vector (EV) or KITENIN‐V5 were transfected with CTD‐HA and treated with KDIP for 24 h. The cells were analysed by the indicated antibodies. (C) Effect of MG132 on the suppression of cell invasiveness by KDIP. Caco2 cells were transfected with EV or KITENIN‐V5 and treated with KDIP (1 μM, 24 h). During this experimental period, cells were treated again with MG132 (10 μM) for 12 h before being harvested and subjected to invasion assay. The pictures and histogram of the invasion assay were obtained as in Figure 2D. (D) Levels of Myo10 and KITENIN are reduced more after treatment with KDIP under Nrdp1 expression. Caco2/KITENIN cells were transfected with EV or Nrdp1 and then treated with KDIP (1 μM). The protein levels of Myo10 and KITENIN were checked after treatment with cycloheximide (CHX, 20 ng/ml) at the indicated times. A significant difference in KITENIN level was observed after treatment with KDIP in the absence (^**^ *p* < .01) or presence (^##^ *p* < .01) of Nrdp1 expression. Asterisks (^$^ *p* < .05) indicate a significant difference between the indicated groups. (E) E3‐ligase Nrdp1 associates with ubiquitination of Myo10 in KDIP‐treated Caco2 cells. Caco2 cells were transfected with KITENIN and Nrdp1 or ubiquitin and treated with scrambled peptide or KDIP (1 μM) for 24 h. The cell products immunoprecipitated with an anti‐Myo10 antibody were separated on 6% SDS–polyacrylamide gels, transferred and immunoblotted with an anti‐ubiquitin antibody. (F) E3‐ligase Nrdp1 mediates the effects of treatment with KDIP on the degradation of Myo10 and decreased cell invasion. Caco2 cells were transfected with EV or KITENIN‐V5 under knockdown of Nrdp1 via siRNA transfection, and treated with KDIP (1 μM) for 24 h. The cells were analysed by immunoblot assay to detect the Myo10 and KITENIN level (left panel) or invasion assay (right panel). The pictures and histogram of the invasion assay were obtained as in Figure 2D (mean ± SEM, *n* = 4, ^*^ *p* < .05, ^**^ *p* < .01, ^***^ *p* < .001). (G) Schematic showing how Myo10 stabilizes the KITENIN homodimer and the effects of KDIP on the disintegration of the KITENIN dimer and its stability. Here, Myo10 acts as an effector that selectively binds to KITENIN and regulates the oncogenic activity of KITENIN by stabilizing its dimerization. Through the downregulation of Myo10, KDIP exhibits more specific anti‐oncogenic activity in cancer cells expressing higher levels of KITENIN. RIM, RACK1‐interacting motif

**Figure:**
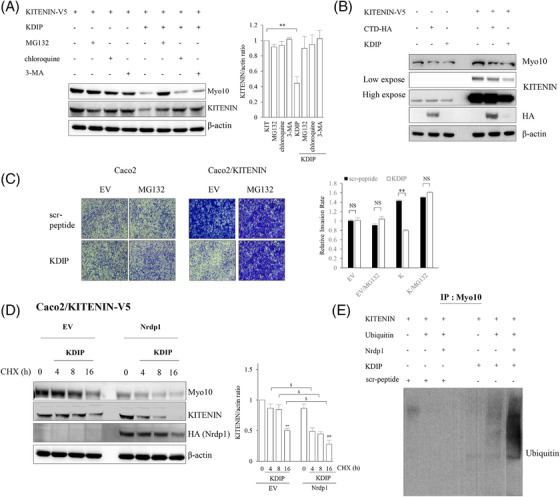

**Figure:**
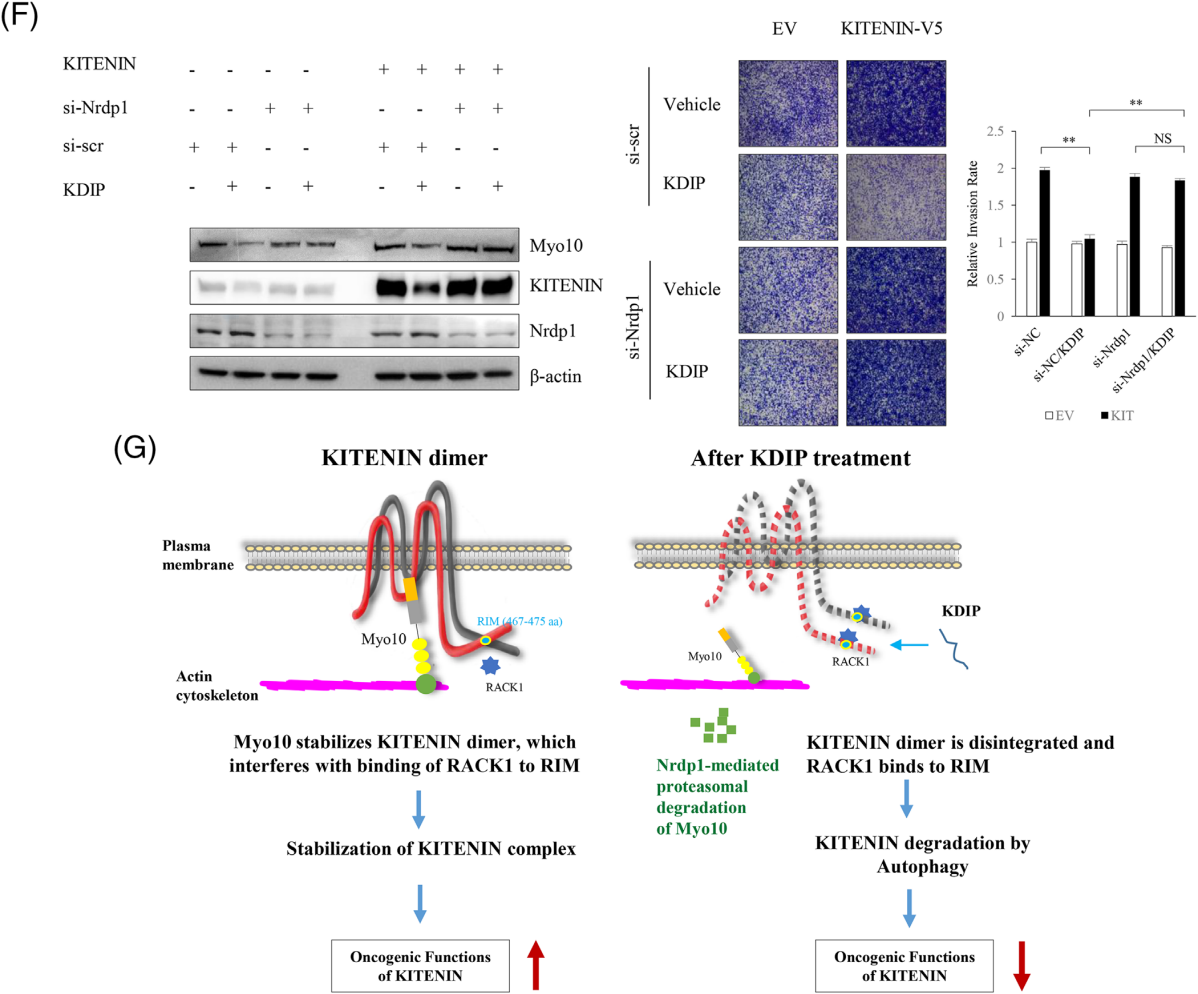

Because E3‐ligase Nrdp1 interacts with KITENIN‐CTD within the functional KITENIN/ErbB4 complex and mediates the proteasomal degradation of KITENIN‐bound Dvl2 to generate c‐Jun in the EGF‐KITENIN/ErbB4‐c‐Jun axis,^39^ we checked whether Nrdp1 is also involved in the KDIP‐induced proteasomal degradation and thereby downregulation of Myo10, which is also bound to the transmembrane portion of KITENIN (Figure 5A). First, to test whether the stability of Myo10 is modulated by E3‐ligase Nrdp1, we monitored the amount of Myo10 after treatment with cycloheximide, a reagent that inhibits protein synthesis, in the presence or absence of Nrdp1. The results revealed that expressions of both KITENIN and Myo10 were decreased after treatment with KDIP. However, when Nrdp1 was transfected under these conditions, the levels of KITENIN and Myo10 were further decreased (Figure 6D). Next, we examined the effect of Nrdp1 on the ubiquitination of Myo10 and found that KDIP‐induced ubiquitination of Myo10 was markedly increased by forced expression of Nrdp1 (Figure 6E). As shown in Figure 6F, those effects observed after treatment with KDIP, such as suppression of increased cell invasion by forced expression of KITENIN as well as the decreased expression of Myo10 and KITENIN, were rescued by knockdown of Nrdp1. These results indicated that the E3‐ligase Nrdp1 is also responsible for the proteasomal degradation of Myo10 after KDIP. We interpreted these data as follows: After treatment with KDIP, first the E3‐ligase Nrdp1‐mediated proteasomal degradation of Myo10 was induced and, second, the downregulated Myo10 resulted in the degradation of KITENIN in a lysosome‐autophagy pathway‐dependent manner.

Collectively, we can summarize these results as follows: Because KITENIN achieves stability by forming dimers in the cell membrane, the oncogenic function of KITENIN will also be an event in the cell membrane. Myo10 stabilized the *cis* form of the KITENIN homodimer by tying up the two intracellular cytoplasmic loops of the transmembrane portion of KITENIN. The downregulation of Myo10 after KDIP via proteasomal degradation led to the further loosening and disintegration of the KITENIN dimer, thus in concert with KDIP to expose the RIM site, which caused an increased interaction of RACK1 with the exposed RIM and thereby triggered the degradation of KITENIN in an autophagy‐dependent manner (Figure 6G).

### 3.9 Treatment with KDIP suppresses the tumour growth and hepatic metastasis of CRC in vivo

We first examined whether KDIP has in vivo anti‐tumour effects by using a syngeneic tumour model in BALB/c mice and the CT‐26 murine colon adenocarcinoma cell line. Before investigating the in vivo effect of KDIP, we tested the effect of KDIP on the in vitro invasiveness of CT‐26 cells. Cell invasion was analysed with CT‐26/KITENIN‐V5 cells that stably expressed KITENIN‐V5 and was significantly reduced by increasing doses of KDIP, compared with that of scramble peptide (Figure 7A).

FIGURE 7Effect of KAI1 C‐terminal interacting tetraspanin (KITENIN) dimerization‐interfering peptide (KDIP) on tumour growth and metastasis in in vivo mouse model of colorectal cancer (CRC). (A) Treatment with KDIP decreases cell invasion of KITENIN‐overexpressing CT‐26 cells. Cell invasion assay was performed in CT‐26 cells transfected with KITENIN and treated with scr‐peptide or KDIP (1 μM) (upper panel). Images represent three independent experiments. The pictures and histogram of the invasion assay were obtained as in Figure 2D. After suspension of 7 × 10^4^ KITENIN‐transfected Caco2 cells (Caco2/KITENIN), or 7 × 10^4^ KITENIN‐transfected CT‐26 cells expressing iRFP (CT‐26/iRFP/KITENIN), or 1×10^5^ HCT116 cells in medium containing .2% BSA, cells were treated with scramble peptide (1 μM) or KDIP for 24 h at the indicated concentrations (up to 10 μM) and subjected to invasion assay (lower panel). Based on these data, the half‐maximal inhibitory concentration (IC_50_) of KDIP on cell invasion was obtained. The asterisk indicates a significant difference in Caco2/KITENIN‐V5 cells (scr‐peptide‐treated vs. KDIP‐treated; ^**^ *p* < .01; ^***^ *p* < .001), or CT‐26/iRFP/KITENIN cells (scr‐peptide‐treated vs. KDIP‐treated; ^#^ *p* < .05; ^##^ *p* < .01), or HCT116 cells (scr‐peptide‐treated vs. KDIP‐treated; ^**^ *p* < .01; ^***^ *p* < .001). (B) The intravenous injection of KDIP significantly reduces the tumour burden in a syngeneic mouse tumour model with the higher levels of KITENIN expression. 1 × 10^6^ CT‐26/KITENIN‐V5 cells were inoculated subcutaneously into BALB/c mice. After tumours grew for 1 week, peptide was given intravenously alternate days for 14 days. The mice were sacrificed on Day 30, and images of the tumours and tumour weights in different treatment groups were obtained (mean ± SEM, middle panel). The line graphs of tumour growth and individual body weights of the CT‐26/KITENIN‐tumour‐mice after intravenous injection with vehicle, scr‐peptide or KDIP (1 and 5 mg/kg, mpk) are represented as mean ± SEM (lower panel). (C) The intravenous injection of KDIP significantly reduces colorectal liver metastasis in an intrasplenic hepatic metastasis model with the higher levels of KITENIN expression. An experimental hepatic metastasis model was prepared by intrasplenic inoculation of stably CT‐26/iRFP/KITENIN‐V5 expressing cells into syngeneic mice and followed by splenectomy. For 2 weeks, mice were injected intravenously with KDIP (5 mg/kg) or scrambled peptide dissolved in .1% DMSO three times/week. For the evaluation of metastasis, total fluorescence emitted from liver nodules expressing iRFP was measured and counted (left panel) or metastatic tumour growth was counted as nodules that migrated to the surface of the liver, and a metastatic score was obtained by multiplying it with size (middle panel). Metastatic score or total fluorescence is represented as mean ± SEM. An asterisk indicates a significant difference between indicated groups (^*^ *p* < .05). The line graphs of individual body weights after intravenous injection with scr‐peptide or KDIP (5 mg/kg, mpk) are represented as mean ± SEM (lower panel). (D) The decreased levels of Myosin‐X (Myo10), KITENIN and its downstream signals, and phospho‐c‐Met, but the restored level of KAI1 in regressed tumour tissues given KDIP. The expression levels of Myo10, eukaryotic elongation factor 2 (eEF2), KITENIN, phospho‐ERK, phospho‐c‐Jun, KAI1 and phospho‐c‐Met were examined via immunoblot analyses in metastatic liver nodules collected from both groups, KDIP (5 mg/kg) versus scrambled peptide (scr‐pep). The quantitative amount of each protein was measured by densitometry and is represented as an arbitrary score (mean ± SEM, *n* = 6). An asterisk indicates a significant difference between indicated groups (^*^ *p* < .05, ^**^ *p* < .01, NS: not significant).

**Figure:**
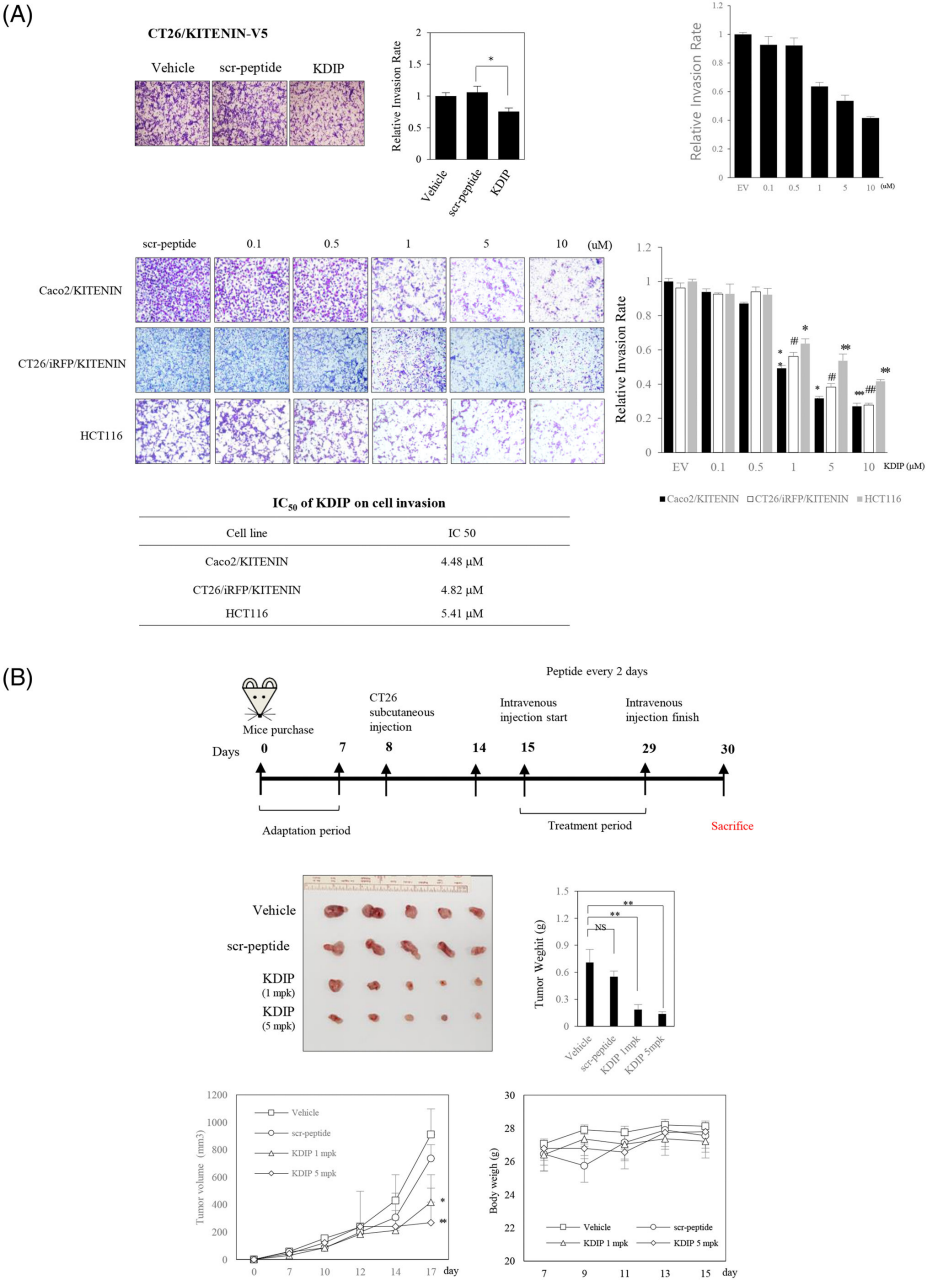

**Figure:**
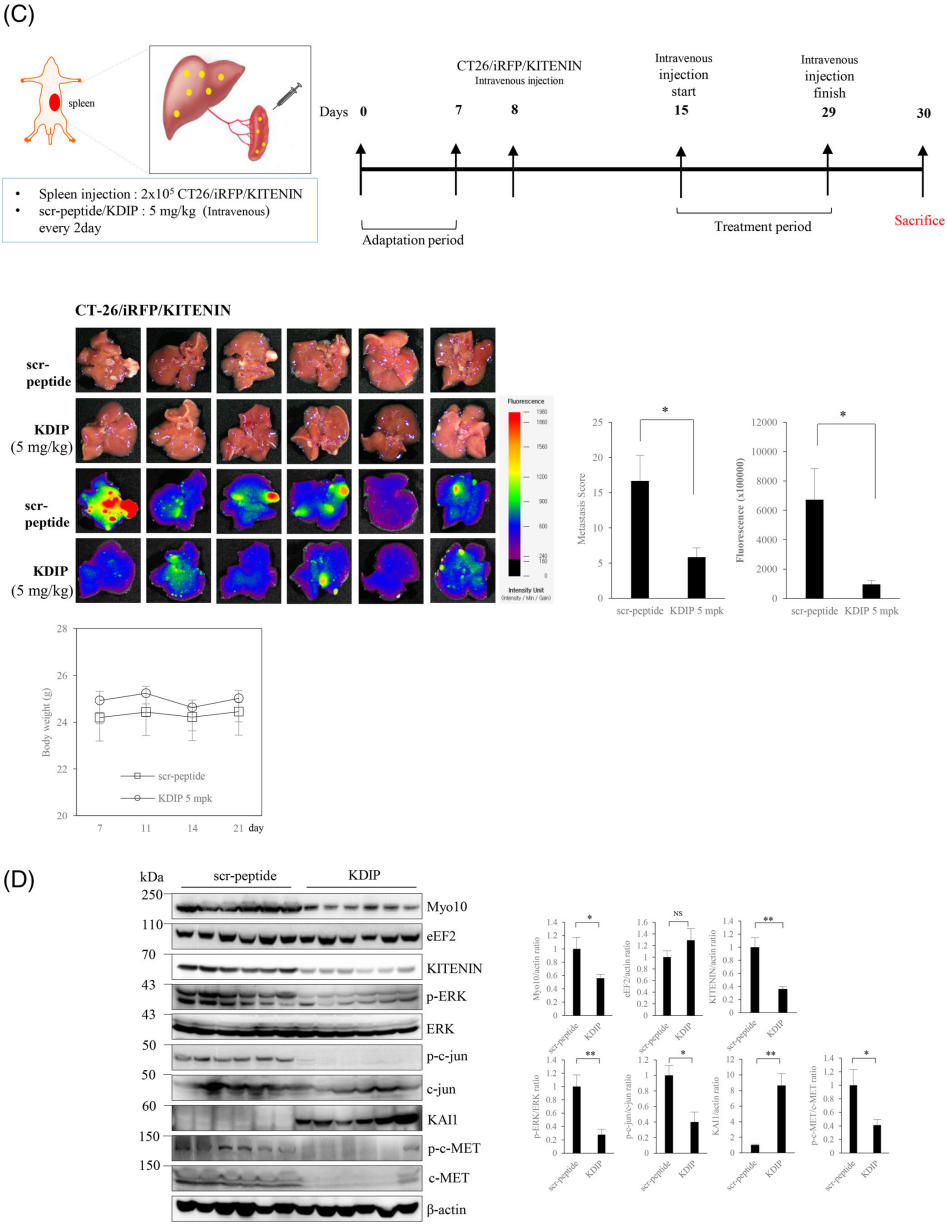

For the in vivo experiments, we explored the pharmacokinetics of the injected peptide. We first examined the in vitro serum disappearance of KDIP in the mouse. When incubated with mouse serum for 20 min, in addition to the original KDIP (20‐mer), the N‐truncated forms of KDIP (15‐mer, 16‐mer, 17‐mer, 18‐mer, 19‐mer) were detected in serum by mass spectrometry (Figure S8A). The peak concentration of KDIP was reached within 5 min and its serum level was maintained around 160 min after administration (Figure S8B). This finding indicated that KDIP was relatively rapidly inactivated by serum in vitro, with a terminal half‐life of 30.9 min.

For the experimental tumour model, CT‐26/EV or CT‐26/KITENIN‐V5 cells were injected into the right back of BALB/c mice to form tumours, and KDIP was injected intravenously according to the timeline shown in Figure 7B. Considering the suppressive effect of KDIP on the cell invasion of CT‐26 cells, the initial test dosage was set to 1 mg/kg, which is equivalent to the half‐maximal inhibitory concentration (IC_50_) of KDIP on cell invasion. The dosage of KDIP was increased to 5 mg/kg in subsequent experiments using CT‐26/KITENIN‐V5 cells. KDIP was administered every other day for 2 weeks via intravenous injection from 7 days after cell injection. After 2 weeks of treatment, the mice were sacrificed and the tumours were analysed.

In experiments using CT‐26/EV cells that are not forced to overexpress KITENIN, tumour growth in the KDIP‐treated group showed a tendency to be slightly reduced at 2 weeks after injection, compared with those of vehicle‐treated or scramble peptide‐treated group (Figure S8C). However, basically, KDIP had little effect on tumour growth in the EV group. However, in experiments using CT‐26/KITENIN‐V5 cells that are forced to overexpress KITENIN, tumour volume was significantly reduced by KDIP, and this effect was dependent on the dosage of KDIP used (Figure 7B). However, the N‐truncated forms of KDIP (17‐mer, 19‐mer) as well as the original KDIP (20‐mer) were not detected by mass spectrometry in regressed tumour tissues or liver (Figure S8E), which were collected after fifth intravenous injection of KDIP (Figure S8D). Although an intravenous administration of KDIP has an evident tumour regressive effect (Figure 7B), this finding from mass spectrometry suggested that the tissue levels of KDIP after intravenous injection, original or truncated forms, were very low, which is below the limit of detection.

We further tested the anti‐tumour effect of KDIP by using a mouse model of liver metastasis of CRC. To create the experimental models, we performed intrasplenic inoculation of stably CT‐26/KITENIN‐iRFP‐expressing cells in syngeneic mice, and the mice were allowed to recover for 2 weeks after subsequent splenectomy for migration of injected CT‐26/iRFP/KITENIN‐V5 cells to the liver through the portal vein. For 2 weeks, the mice were injected intravenously with KDIP every other day. Liver metastasis was evaluated by detecting total fluorescence emitted from tumour nodules expressing iRFP. The fluorescence of the hepatic nodule was significantly reduced by increasing doses of KDIP to 5 mg/kg, compared with that of the scramble peptide group (Figure 7C). Also, we analysed the expression levels of KITENIN, Myo10 and eEF2 in the metastatic liver nodules collected from both groups and found that the protein level of Myo10, but not of eEF2, was significantly decreased by KDIP (Figure 7D). As shown in Figure 7D, KITENIN and its downstream signals,^19^, ^21^ such as phospho‐ERK and phospho‐c‐Jun, were significantly decreased by KDIP. Because we reported that KITENIN expression is inversely correlated with the expression of KAI1 and other metastasis suppressor genes,^18^ we compared the expression levels of KITENIN and KAI1 in the metastatic liver nodules collected from both groups. We found that the decreased expression of KAI1 in the scr‐peptide‐injected group was significantly restored in the KDIP‐injected group with the degradation of KITENIN (Figure 7D). In addition, the expression of phospho‐c‐MET was significantly decreased in the KDIP‐injected group compared with the scr‐peptide‐injected group (Figure 7D).

These results positively strengthened the in vitro result showing the inhibition of the oncogenic function of KITENIN by KDIP. Overall, KDIP effectively inhibits the oncogenic activity by KITENIN through a specific inhibition of the dimerization of KITENIN.

### 3.10 A positive correlation exists between the expression levels of *KITENIN* and *Myo10* in colorectal adenocarcinoma in TCGA

To assess quantitatively the relationship between the expression levels of *KITENIN* and *Myo10* in CRC patients, we calculated a correlation score by using TCGA. We found a positive correlation (*r* = .222 in PanCancer datasheet; *r* = .318 in Colorectal Cancer project, ^*^ *p* < .001) between *KITENIN* and *Myo10* expression in colorectal adenocarcinoma in TCGA (Figure S9). In addition, the score was slightly higher in tumour tissues from stage IV CRC (*r* = .649 in Colorectal Cancer project; *r* = .240 in PanCancer datasheet) than in tumour tissues from other CRC stages. These results suggest the expression levels of *KITENIN* and *Myo10* as a prognostic indicator of advanced CRC stage.

## 4 DISCUSSION

Peptides have low molecular weights and good cellular uptake and can specifically bind to tumour cells with low toxicity to normal tissues. Accordingly, they are ideal molecules for targeted cancer therapies.^40^ Similar to antibodies, specific peptides not only bind to target proteins but also block their signalling and function.^41^ In this study, we introduced KDIP, a short peptide that effectively suppressed oncogenic KITENIN in vitro and in vivo through specific inhibition of the dimerization of KITENIN and by reducing the stability of the KITENIN protein. Adding to reports of siRNA,^25^ mi‐RNA^26^ and small‐molecular‐weight compounds^27^ targeting the functional KITENIN complex, our present result is the first report on the inhibition of the oncogenic function of KITENIN by a peptide.

RACK1 was previously identified as a novel hypoxia‐inducible factor‐1α (HIF‐1α)‐interacting protein that regulates HIF‐1α stability through competition with HSP90 and recruitment of the Elongin‐C/B ubiquitin ligase complex.^42^ Likewise, in this study, we found that the binding of RACK1 to aa 463–471 of KITENIN (RIM) is essential to the degradation of KITENIN. The formation of a stable KITENIN homodimer via the tying up of two intracellular cytoplasmic loops of the transmembrane portion might create a structural hindrance to mask RIM from binding with RACK1. Thus, just as treatment with KDIP caused the downregulation of Myo10 and induced the loosening and disintegration of the KITENIN dimer to expose the RIM site, it also led to an increased interaction of RACK1 with the exposed RIM. This increased interaction thereby triggered the degradation of KITENIN in an autophagy‐dependent manner and reduction of the KITENIN dimer. Thus, on the basis of the present results and previous observations that KITENIN does not have receptor function,^18^, ^19^, ^21^ we propose that the KITENIN homodimer is actually a structural and functional unit for the oncogenic function of KITENIN. When KITENIN acts as an enhancer of invasion in CRC cells, it serves as a scaffolding protein to form the functional KITENIN complex in which the KITENIN homodimer offers benefits, such as structural hindrance to binding with RACK1 or to interaction of Nrdp1 with Myo10, which thereby protects the protein from degradation.

Myo10 is involved in cell–cell adhesion‐associated signalling and cytoskeleton reorganization.^35^, ^43^ Its protein structure, an anti‐parallel coiled‐coil, is optimized for movement on actin bundles.^44^ This characteristic is critical for the role of Myo10 as a fast filopodial transporter and perhaps for its role as an actin organizer to promote filopodia formation.^45^, ^46^ Myo10 and Myo1b are both expressed at higher levels in prostate cancer cells with high metastatic potential and in metastatic prostate cancer tissues.^47^ Myo10 knockdown ablates filopodia and decreases the speed at which filopodia migrate. Myo1b knockdown, by contrast, increases the numbers of stress fibres but does not affect the speed of migration, indicating that these molecular motor myosins use actin as ‘tracks’ to walk along but also directly influence actin organization and cell morphology, which can contribute to the metastatic phenotype. Myo10 was also reported to colocalize with vascular endothelial (VE)‐cadherin in filopodia and to move synchronously with it, indicating that Myo10 establishes a link between the actin cytoskeleton and VE‐cadherin, thereby allowing VE‐cadherin transportation along intrafilopodial actin cables.^48^ Thus, it was proposed that the trafficking of VE‐cadherin along filopodia using Myo10 is a prerequisite for the formation of cell–cell junctions, a process that may be of functional importance in endothelium repair and angiogenesis. Our present results showing an interaction of Myo10 with the transmembrane portion of KITENIN suggest that Myo10 establishes a link between the actin cytoskeleton and KITENIN, an atypical tetraspanin, thereby allowing anchoring and transportation of KITENIN on the plasma membrane. Therefore, the stabilization of the KITENIN homodimer by Myo10 is a prerequisite for the formation of the oncogenic KITENIN complex, which triggers a downstream signalling process for increased cell invasiveness and spread of metastasis.

It is well established that tetraspanin interacts and collaborates with other membrane proteins, including other tetraspanins, growth factor receptors and integrins at tetraspanin‐enriched microdomains (TEMs) to regulate multiple stage of cancer development.^49^, ^50^ In some cases, such as CD9, CD81 and CD151, it exists as a homodimer, which may be a basic structural unit.^49^ In addition, associations of tetraspanin within the TEM are classified into three interactions.^51^ The first is primary interaction: relatively direct and strong interactions between tetraspanins that are resistant or partially resistant to relatively stringent detergents,^52^, ^53^ such as 1% Triton X‐100 and digitonin; the second is the weaker interaction between different primary complexes that are preserved in less stringent detergent,^54^ such as 1% Brij97, but not in more harsh detergent; the third is relatively weak interaction that is preserved in mild detergents,^54^ such as CHAPS, Brij58 and Brij98, but not in 1% Triton X‐100 and Brij97. In accordance with this classification and our present data of the preservation of the KITENIN homodimer under harsh denaturation and reducing conditions, the KITENIN dimer is thought to be derived from a direct and strong primary interaction.

KITENIN is an aggressive agent in the progression of various human cancers; we have shown that the expression of KITENIN is significantly higher in human colon,^19^, ^20^ laryngeal,^55^ oral cavity squamous,^56^ gastric,^57^ hepatocellular^58^ and glioma^59^ tumour tissues than in corresponding normal mucosa. In this regard, KITENIN is a promising target for anti‐metastasis therapy.^27^ As shown in Figures 6G and S3A, KDIP had a specific effect on the KITENIN dimer and inhibited the downstream signalling of the KITENIN complex. After treatment with KDIP, Myo10 was decreased via proteasomal degradation and thereby the KITENIN dimer lost its stability. Binding of RACK1 to RIM caused a degradation of KITENIN in an autophagy‐dependent manner. These results indicated that the downregulation of Myo10 is responsible for the therapeutic action of KDIP on colorectal tumours with higher KITENIN expression through a disintegration of the KITENIN dimer and thereby a degradation of KITENIN protein. Also, this peptide efficiently inhibited the hepatic metastasis of CRC tumour xenografts and orthotopic CRC tumour in syngeneic mice. Interestingly, as in the cultured CRC cells in which the overexpression of KITENIN resulted in a reduced expression of KAI1 and vice versa,^18^ the decreased expression of KAI1 in the metastatic liver nodules collected from the control group was restored in samples from the KDIP‐injected group with the degradation of KITENIN (Figure 7D). Additionally, as in the KAI1 re‐expressing PC3 prostate cancer cells that showed a suppression of both integrin‐ and ligand‐mediated activation of c‐Met,^60^ the expression of phospho‐c‐MET was also decreased in the KDIP‐injected metastatic liver nodules (Figure 7D). These expressional changes of KAI1 and c‐MET after treatment with KDIP may have contributed to the inhibition of colorectal liver metastasis by KDIP. Thus, KDIP represents a new agent that could be used in combination with existing anticancer therapeutics to block the oncogenesis of CRC expressing higher levels of KITENIN.

In breast carcinomas, Myo10 is predominantly expressed at the invasive edges of the carcinoma, and its expression is correlated with the presence of TP53 mutations and poor prognosis. Furthermore, the transport of β1 integrins to the filopodia tip by Myo10 is required for cell invasion.^61^ These findings suggest that, in addition to prostate cancer cells, Myo10 is required for breast cancer cell invasion. In addition, Myo10 was shown to exert a direct effect on invadopodia to promote the invasive growth of breast tumour and metastasis in a nude mouse model^62^ but also participated in lung adenocarcinoma metastasis.^63^ In this study, we found that the expression of Myo10 was increased in KITENIN‐transfected CRC cells and that Myo10 further stabilized the KITENIN dimer. We also found a positive correlation between *KITENIN* and *Myo10* expression in colorectal adenocarcinoma in TCGA. These reports and our present data suggest that in KITENIN‐overexpressing cancer tissues, the upregulation of Myo10 and resultant stabilization of overexpressed KITENIN contribute to the aggressive invasiveness and metastasis in mutant p53‐driven cancers. Both proteins might serve as specialized metastatic engines to establish an invasive cellular phenotype, which could be seeded on supportive soil promoting metastasis.^64^ Because the downregulation of Myo10 is responsible for the therapeutic action of KDIP on colorectal tumours with higher KITENIN expression, we propose that KDIP could be used as a dual blocker to the oncogenic function of both KITENIN and Myo10 proteins.

In summary, an essential function of the KITENIN homodimer is maintaining its stability and thereby providing a docking site for key interaction partners, and our findings propose a new strategy for developing a peptide cancer therapeutic to specifically block the colorectal liver metastasis driven by the higher levels of oncogenic KITENIN.

## CONFLICT OF INTEREST

The authors declare that there is no conflict of interest.

## Supporting information

Supporting Information

Supporting FigureS1 Information

Supporting FigureS2 Information

Supporting FigureS3 Information

Supporting FigureS4 Information

Supporting FigureS5 Information

Supporting FigureS6 Information

Supporting FigureS7 Information

Supporting FigureS8‐1 Information

Supporting FigureS8‐2 Information

Supporting FigureS9 Information
