# Supplementary material for: A peptide interfering with the dimerization of oncogenic KITENIN protein and its stability suppresses colorectal tumour progression
Source: Clin Transl Med. 2022 Jul 19;12(7):e871. doi: 10.1002/ctm2.871 (PMC9296036; doi:10.1002/ctm2.871)
Supplement: Supplementary file 1 — Supporting Information [file CTM2-12-e871-s001.docx]

**Supporting Information**

**Supplementary Figure Legends**

**FIGURE S1** KITENIN forms a homodimer in the *cis* formation. A, KITENIN forms homodimers in co-transfected CRC cells. Caco2 and HCT116 cells were transfected with both KITENIN-V5 and KITENIN-myc plasmids. After 48 h of incubation, immunoprecipitation was performed with anti-V5 or anti-Myc antibody, respectively, and analyzed via immunoblotting with the indicated antibodies. The IgG band was used as a control for the antibody dose in the immunoprecipitation analyses. Each protein level was examined through immunoblot analyses by using whole-cell lysates (WCL). B, Presence of an endogenous KITENIN dimer in non-transfected CRC cells. WCL (80 μg) prepared by using IP cell lysis buffer from HCT116 cells were run on a regular SDS-PAGE gel in the reducing/denaturing condition and immunoblotted with an anti-KITENIN antibody (left panel). WCL (20 μg) from empty vector (EV)- or KITENIN-transfected Caco2 cells was served as a positive control. Protein size is indicated by an arrowhead. Intensities of the 130-kDa KITENIN homodimer band (red arrowhead) and the 70-kDa KITENIN monomer band (blue arrowhead) were compared in low or high expose state. Cropped images from a whole SDS-PAGE gel were shown (right panel). It showed the comparative size of the KITENIN dimer. C, Schematic diagram representing the possible model of the homodimerization of KITENIN. D, KITENIN homodimers exist exclusively in the *in cis* form. HEK-293T cells were transfected with plasmids encoding KITENIN-Myc or KITENIN-V5 as follows: cells were transfected with empty vector (lane 1), co-transfected with both plasmids KITENIN-Myc and KITENIN-V5 (lane 2), transfected with each plasmid alone but mixed for co-culture (lane 3), transfected with KITENIN-Myc (lane 4), or transfected with KITENIN-V5 (lane 5). The cells were immunoprecipitated with anti-Myc or anti-V5 antibody, respectively, and analyzed by the indicated antibodies. The co-cultured condition (lane 3) was served as a negative control for the *cis* form and interactions were not found in co-cultured cells.

**FIGURE S2** Inhibition of KITENIN dimerization and suppression of cell motility by the defined intracellular C-terminal regions of KITENIN. A, Schematic depiction of deletion mutants of KITENIN and effects of the defined intracellular C-terminal regions of KITENIN on dimerization of KITENIN. Intracellular KITENIN C-terminal domains were serially deleted at intervals of approximately 40 amino acids from the C-terminal end (524 aa) (left panel). Caco2 cells co-expressing KITENIN-Myc and KITENIN-V5 were transfected with empty vector (EV), or HA-tagged KITENIN constructs, such as wild-type KITENIN (WT), CTD, and serially deleted KITENIN mutants (1-339, 1-394, 1-449, 1-487). Each cell lysate was immunoprecipitated with an anti-V5 antibody and then immunoblotted with an anti-Myc antibody to detect the KITENIN dimer. Numerals indicated a quantification of the MYC bands normalized to the corresponding V5 bands (right panel). B, Effects of the defined intracellular C-terminal regions of KITENIN on cell motility. The Caco2/KITENIN-V5 cells were transfected with HA-tagged deletion mutants of KITENIN (WT, CTD, 1-339, 1-394, 1-449, 1-487) for 48 h and subjected to invasion assay. The pictures and histogram of the invasion assay were obtained as in Figure 2D (mean ± SEM, n=3, **P<0.01, ***P<0.001). C, Design of the stapled KITENIN-targeting peptide. The 7 classified peptide sequences within the C-terminal region (449-487) of KITENIN are listed. D, The 463-471 peptide was the most effective for inhibiting KITENIN dimerization and suppressing cell motility among the 7 classified peptides. Caco2 cells co-expressing KITENIN-Myc and KITENIN-V5 were transfected with empty vector (EV), CTD, or each of 7 classified peptides. Cells were immunoprecipitated with an anti-V5 antibody and then immunoblotted with an anti-Myc antibody (left panel) or subjected to invasion assay (right panel). Numerals indicated a quantification of the MYC bands normalized to the corresponding V5 bands (left panel). The pictures and histogram of the invasion assay were obtained as in Figure 2D (mean ± SEM, n=3, **P<0.01, ***P< 0.001).

**FIGURE S3** Suppression of the oncogenic function of KITENIN by KITENIN 9-mer sequence (463-471 peptide) bearing various cell-penetrating peptides and effect of expression of KITENIN-CTD on KITENIN transcripts. A, Design of variant 463-471 peptides bearing various cell-penetrating or tumor-homing peptides. B, Comparison of the inhibitory effects on dimerization of KITENIN among the variant 463-471 peptides. Caco2 cells co-expressing KITENIN-Myc and KITENIN-V5 were treated with the indicated variant 463-471 peptide for 24 h. Each cell lysate was immunoprecipitated with an anti-V5 antibody and then immunoblotted with an anti-Myc antibody to detect the KITENIN dimer. C, Comparison of the suppressive effects on cell motility among the variant 463-471 peptides. Caco2/KITENIN-V5 cells were transfected with KITENIN-Myc. After 24 h of incubation, cells were treated with the indicated variant 463-471 peptide for 24 h and subjected to invasion assay. The pictures and histogram of the invasion assay were obtained as in Figure 2D (mean ± SEM, n=3, **P<0.01, ***P<0.001). D, Similar levels of KITENIN transcripts after the forced expression of KITENIN-WT, KITENIN-CTD, or KITENIN-NTD. Caco2 cells were transfected with empty vector, WT KITENIN-HA, KITENIN-NTD-HA (1-240 aa), or KITENIN-CTD-HA (110-524 aa). After 48 h, cells were subjected to RT-PCR analysis of KITENIN transcript.

**FIGURE S4** Delineation of specific binding of KDIP to the KITENIN by using grating-coupled interferometry (GCI). A, Schematic overview of the GCI experiments. After direct amine coupling of anti-GST antibody to PCP chips, capturing of GST-alone (FC2), or WT-KITENNIN (FC3), or KITENNIN-CTD (FC4) was followed until the desired density was reached. Various concentrations of KDIP were injected for kinetics analysis for 125, 62.5, 31.25 and 15.625 nM. PBS-P+ running buffer and GST-alone were used for adjustment. B, Purified GST-fusion proteins. Arrows indicate GST alone (~26 kDa), GST-wt-KITENIN (~100 kDa), or GST-KITENIN-CTD band (~55 kDa). C, In FC2, GST-alone was captured at 855.8 pg/mm^2^ and multi-cycle kinetics was performed and adjusted with FC1, which captured a vehicle and used as a reference channel. D, In FC3, WT-KITENNIN was captured at 2491.9 pg/mm^2^ and multi-cycle kinetics was performed and adjusted with FC2 as a reference channel. E, In FC4, KITENNIN-CTD was captured at 2435.1 pg/mm^2^ and multi-cycle kinetics was performed and adjusted with FC2 as a reference channel.

**FIGURE S5** RACK1 binds to endogenous KITENIN and KDIP stimulates the degradation of endogenous KITENIN in non-transfected CRC cells by binding of RACK1 to KITENIN. A, RACK1 specifically binds to endogenous KITENIN. HCT116 cells were immunoprecipitated with an anti-RACK1 antibody and immunoblotted with an anti-KITENIN antibody to detect endogenous KITENIN bound with RACK1. B, HCT116 (upper) or CT-26 (lower) cells were transfected with RACK-GFP. After 24 h, cells were treated with KDIP (1 μM) for 24 h and analyzed by immunoblotting with the indicated antibody. C, HCT116 cells were transfected with KITENIN-V5, or Δ463-471-KITENIN-V5 and/or RACK1-GFP, for 48 h and immunoprecipitated with anti-GFP antibody to detect whether Δ463-471 KITENIN interacts with RACK1. The proteins in whole-cell lysate (WCL) were immunoblotted with the indicated antibody.

**FIGURE S6** Expression of KITENIN-CTD stimulates the degradation of KITENIN through the increased interaction of RACK1 with KITENIN. A-B, RACK1 influences the effects of KITENIN-CTD on degradation of KITENIN (A) and inhibition of cell motility (B). Caco2 cells were co-transfected with WT KITENIN-V5, KITENIN-CTD-HA, and/or RACK1-GFP for 48 h, and analyzed via immunoblotting with the indicated antibody (left, upper) or subjected to invasion assay (left, lower). In another set, Caco2 cells were co-transfected with WT KITENIN-V5, KITENIN-CTD-HA, and/or RACK1-siRNA for 48 h, and analyzed via immunoblotting with the indicated antibody (right, upper) or subjected to invasion assay (right, lower). The pictures and histogram of the invasion assay were obtained as in Figure 2D (mean ± SEM, n=3, **P<0.01). C, Degradation of KITENIN in a lysosome-autophagy pathway-dependent manner after expression of KITENIN-CTD. Caco2 cells were transfected with empty vector or co-transfected with KITENIN and/or KITENIN-CTD for 48 h. During this experimental period, cells were treated again with MG132 (10 μM) and A1 (Bafilomycin A1, 1 μM), CQ (chloroquine, 100 μM), or 3-MA (1 mM) for 4 and 12 h, respectively, before the cells were harvested. The amount of KITENIN protein was checked by an anti-KITENIN antibody. D, Myo10 also acts as a modulator of the endogenous KITENIN in non-transfected cells. HCT116 or CT-26 cells were transfected with si-KITENIN or si-Myo10 for 48 h and analyzed by immunoblot analyses with the indicated antibodies. The expression of Myo10 in HCT116 or CT-26 cells was not affected by knockdown of KITENIN (left panel), but the expression of endogenous KITENIN in HCT116 or CT-26 cells was decreased under knockdown of Myo10 (right panel).

**FIGURE S7** The C-terminal end region of KITENIN is necessary for the KDIP to bind with the KITENIN and to represent its action. A, Caco2 cells were transfected with HA-tagged KITENIN constructs, such as wild-type KITENIN (WT) and serially deleted KITENIN mutants (1-339, 1-394, 1-449, 1-487), and treated with or without KDIP for 12 h. Each cell lysate was immunoblotted with an anti-HA antibody to detect the HA-tagged KITENIN. Numerals indicated a quantification of the HA-KITENIN bands with or without KDIP treatment, which were normalized to the corresponding actin bands. B, Caco2 cells were transfected with HA-tagged mutant KITENIN construct (1-487), and treated with or without KDIP for 12 h. Cell lysates were run on a native SDS-PAGE gel in the non-reducing/non-denaturing condition and immunoblotted with an anti-HA antibody to detect the HA-tagged KITENIN monomer/dimer. Protein size is indicated by an arrowhead. Intensities of the 130-kDa KITENIN homodimer band and the 70-kDa KITENIN monomer band were compared in low or high expose state. Numerals indicated a quantification of the bands observed.

**FIGURE S8** The pharmacokinetic parameters of the KDIP and effect of KDIP on tumor growth in a syngeneic mouse tumor model that are not forced to overexpress KITENIN. A, Comparison of mass spectrum of KDIP in serum. Upper and lower column corresponded to spectrum obtained after incubation with serum (0 and 20 minutes, respectively). The peaks in mass spectra shown with green or red squares represented the original KDIP (20-mer) or truncated forms of KDIP, respectively (left). The corresponding peptide matching the peak in mass spectra was listed, which were assumed to be derived from the breakdown of KDIP in serum (right). B, The *in vitro* serum disappearance of KDIP in the mouse. The stability of KDIP in serum was examined by mass spectrometry with seven different incubation times at room temperature (0, 5, 10, 20, 40, 80, 160 min). Concentration of KDIP (nM) in serum depending on incubation time was used for plotting (Mean ± SEM, n=3) and the terminal half-life was determined to be 30.86 minutes. C, KDIP had little effect on tumor growth in a syngeneic mouse tumor model using the CT-26 cells without KITENIN overexpression. 3×10^6^ empty vector (EV)-transfected CT-26 (CT-26/EV) cells were inoculated subcutaneously into of BALB/c mice. After tumors grew for 1 week, peptide was given intravenously alternate days for 14 days. The mice were sacrificed on day 30, and images of the tumors (lower panel, left) and tumor weights (mean ± SEM, n=6, lower panel, middle) in different treatment groups were obtained as in Figure 7B. The line graphs of tumor growth (upper panel) and individual body weights (lower panel, right) of the CT-26/EV cells-tumor-mice after intravenous injection with vehicle, scr-peptide, or KDIP (1 mg/kg, mpk) are represented as mean ± SEM (n=6). D, The experiments for the detection of KDIP in tumor tissues from a syngeneic mouse model. 1×10^6^ CT-26/KITENIN-V5 cells were inoculated subcutaneously into of BALB/c mice. After tumors grew for 1 week, peptide was given intravenously alternate days for five times. The mice were sacrificed on day 25, and images of the tumors (lower panel, left) and tumor weights (mean ± SEM, n=4, lower panel, right) in different treatment groups were obtained as in Figure 7B. The line graphs of tumor growth (upper panel) of the CT-26/KITENIN-tumor-mice after intravenous injection with vehicle or KDIP (5 mg/kg, mpk) are represented as mean ± SEM (n=4). E, Comparison of mass spectrum of KDIP in tumor and liver tissues. Representative multiple reaction monitoring (MRM) chromatograms of KDIP were shown. The MRM of original KDIP (501.1 m/z) and truncated KDIP (468.5 m/z and 528.45 m/z) in tumor or liver tissues were compared after administration of vehicle or KDIP.

**FIGURE S9** The expression of *KITENIN* is positively correlated with that of *Myo10* in colorectal adenocarcinoma in TCGA. Correlation between expression of *KITENIN* and *Myo10* was obtained from The Cancer Genome Atlas (TCGA) datasheet based on the PanCancer (A) and Colorectal Cancer project (B). The correlation coefficient *r* values and their significance (*P* values) were calculated through Pearson correlation analysis. RSEM, RNA-seq by expectation maximization; RPKM, reads per kilobase of exon model per million reads.
