# Supplementary figures and images for: A peptide interfering with the dimerization of oncogenic KITENIN protein and its stability suppresses colorectal tumour progression
Source: Clin Transl Med. 2022 Jul 19;12(7):e871. doi: 10.1002/ctm2.871 (PMC9296036; doi:10.1002/ctm2.871)

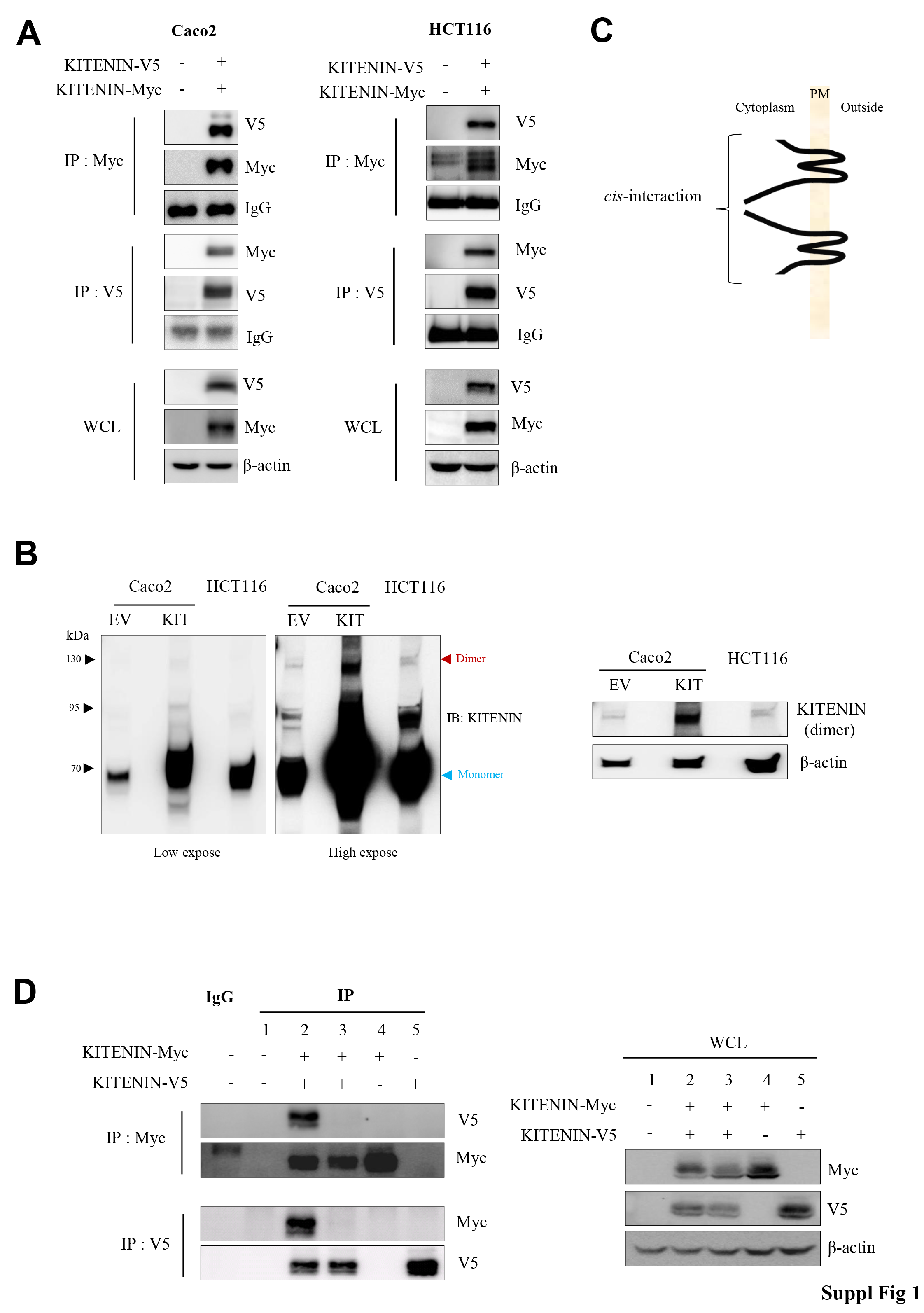

Supplement: Supplementary file 2 — Supporting FigureS1 Information [file CTM2-12-e871-s006.tif]

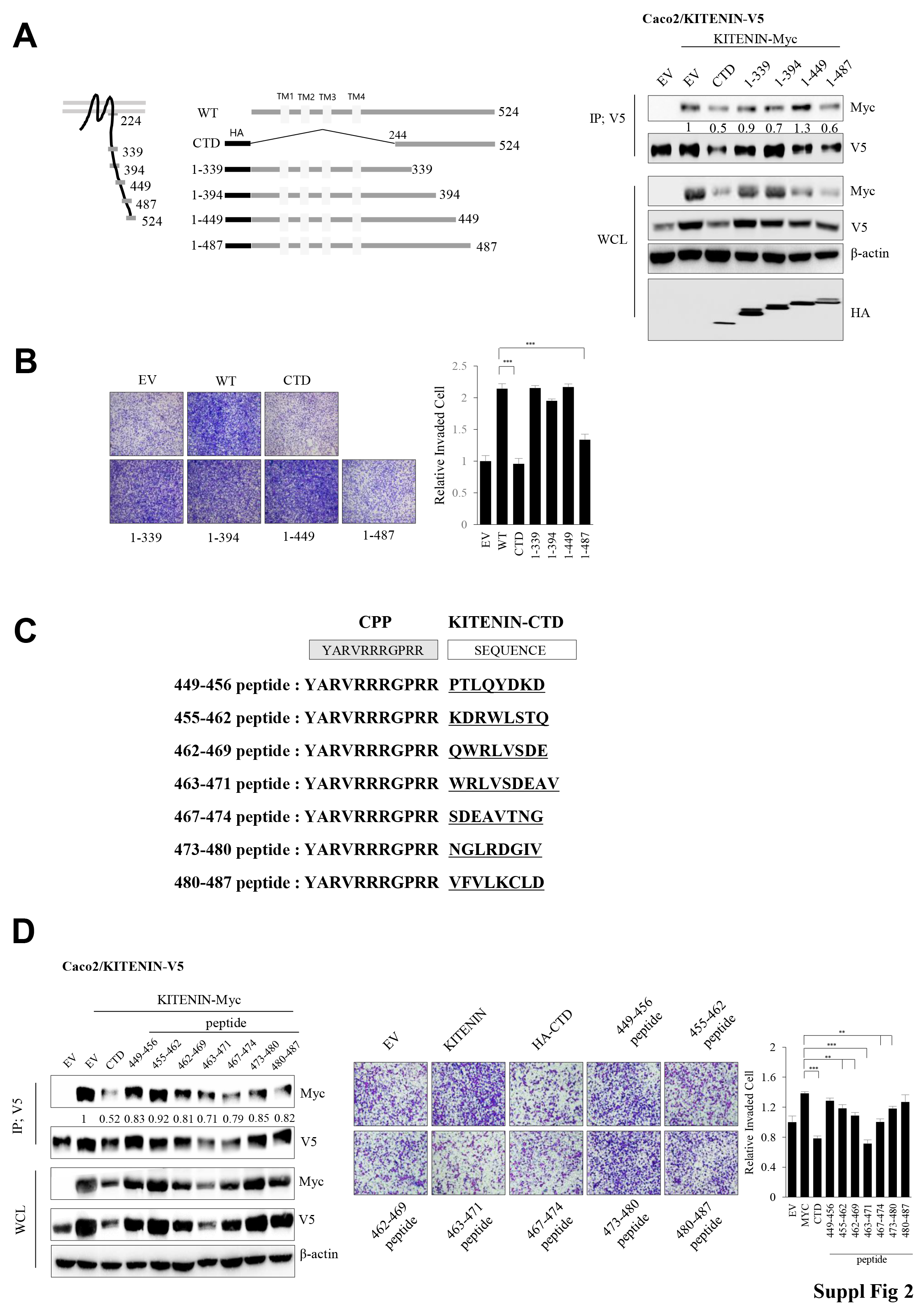

Supplement: Supplementary file 3 — Supporting FigureS2 Information [file CTM2-12-e871-s011.tif]

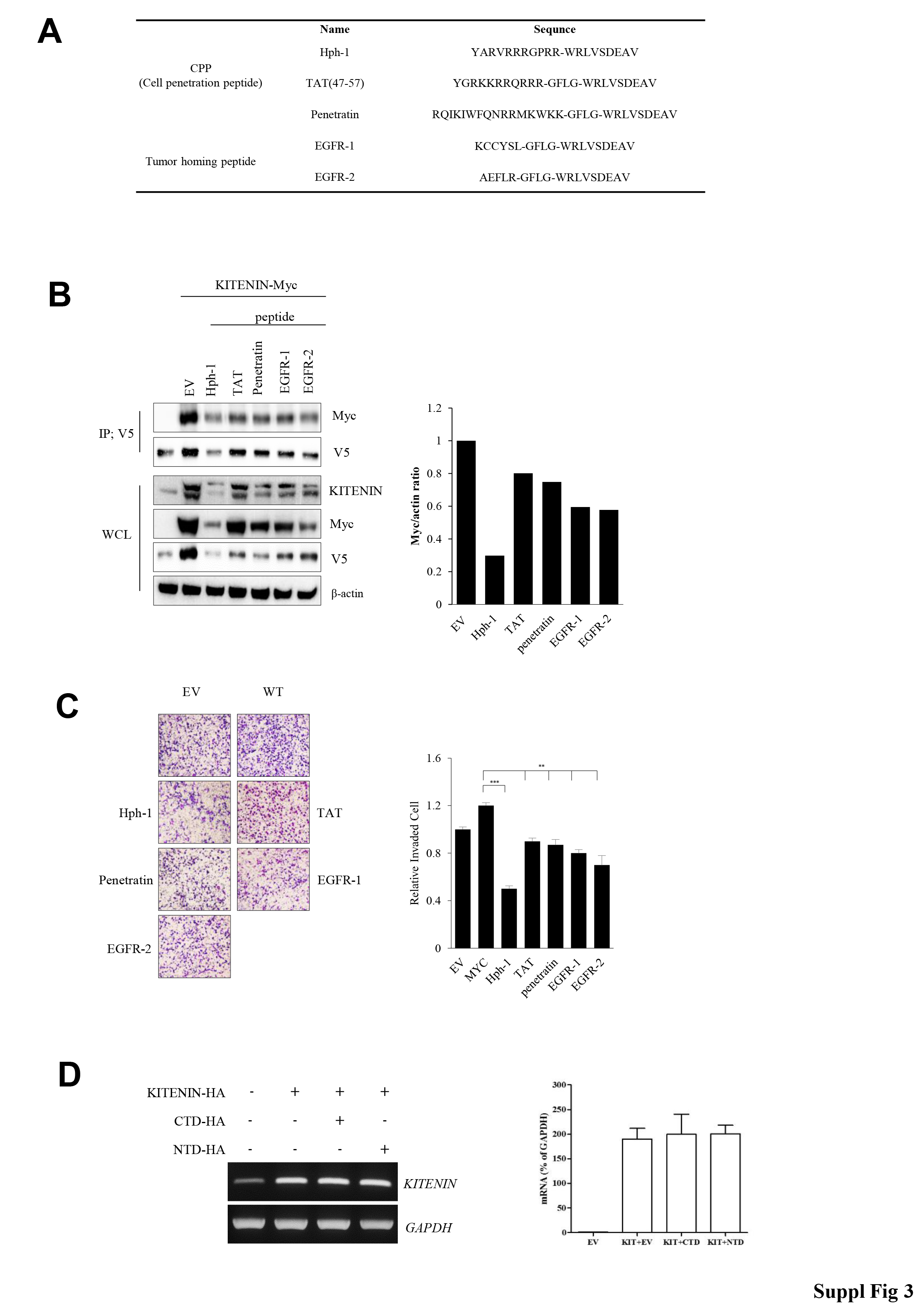

Supplement: Supplementary file 4 — Supporting FigureS3 Information [file CTM2-12-e871-s003.tif]

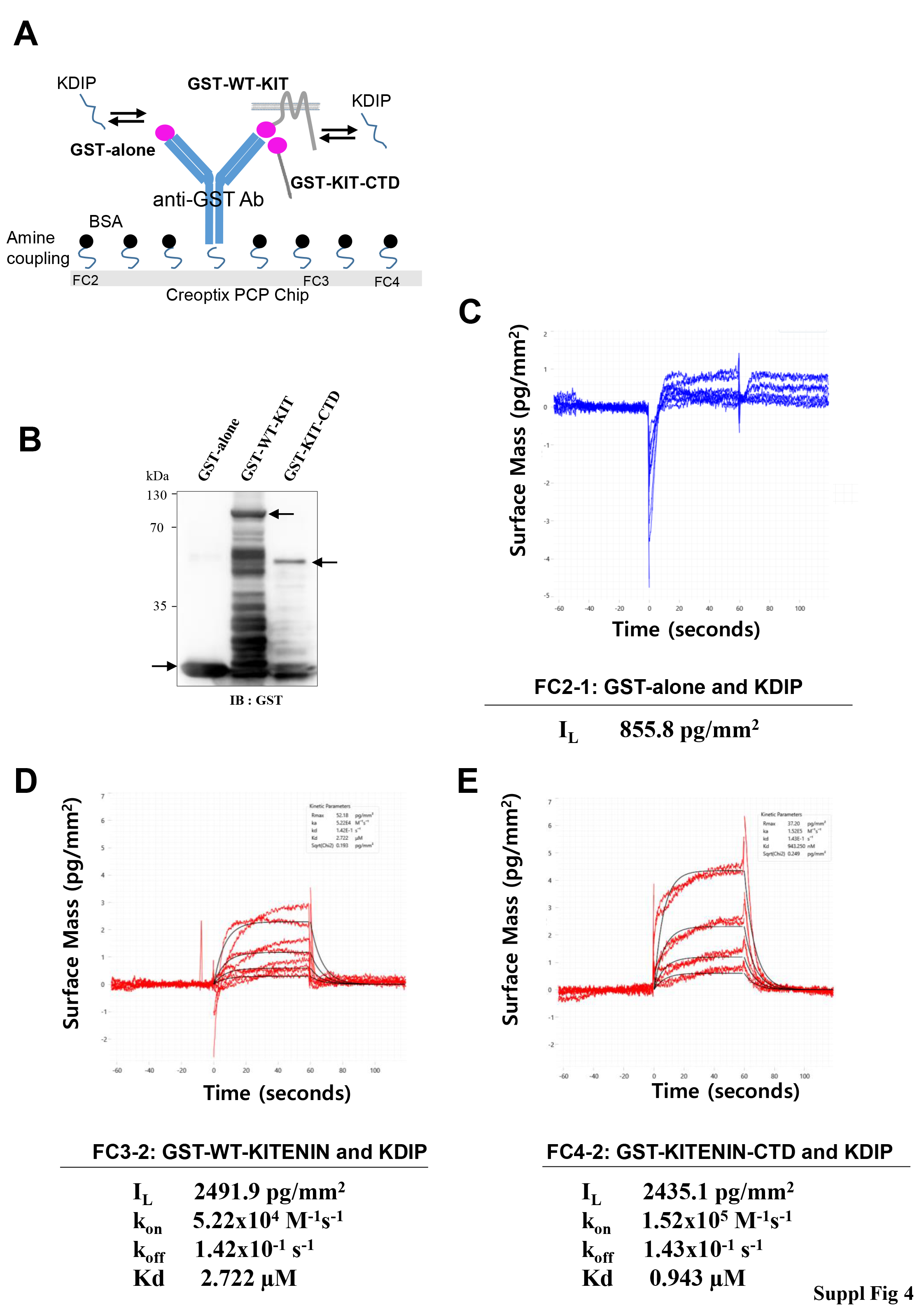

Supplement: Supplementary file 5 — Supporting FigureS4 Information [file CTM2-12-e871-s004.tif]

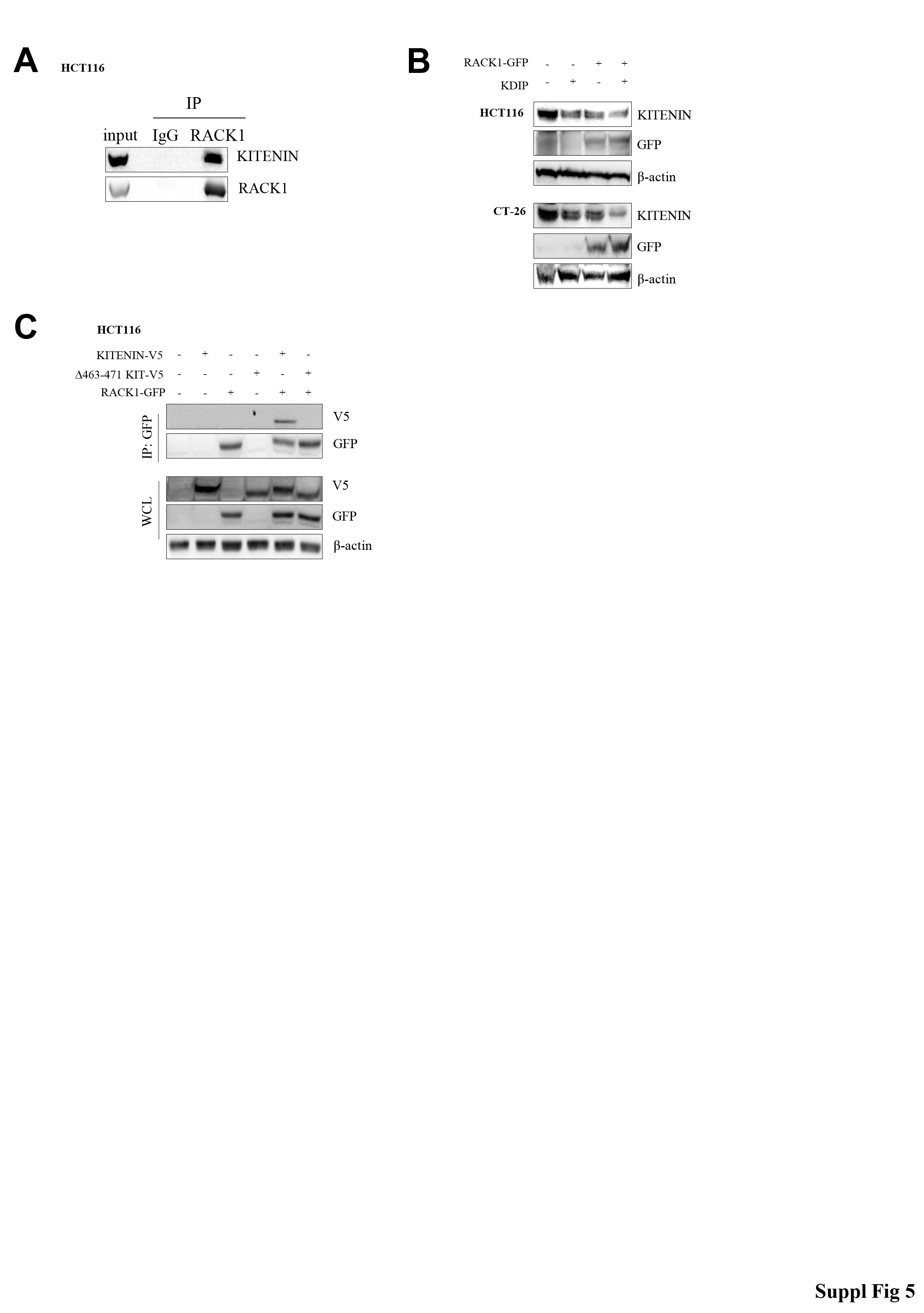

Supplement: Supplementary file 6 — Supporting FigureS5 Information [file CTM2-12-e871-s010.tif]

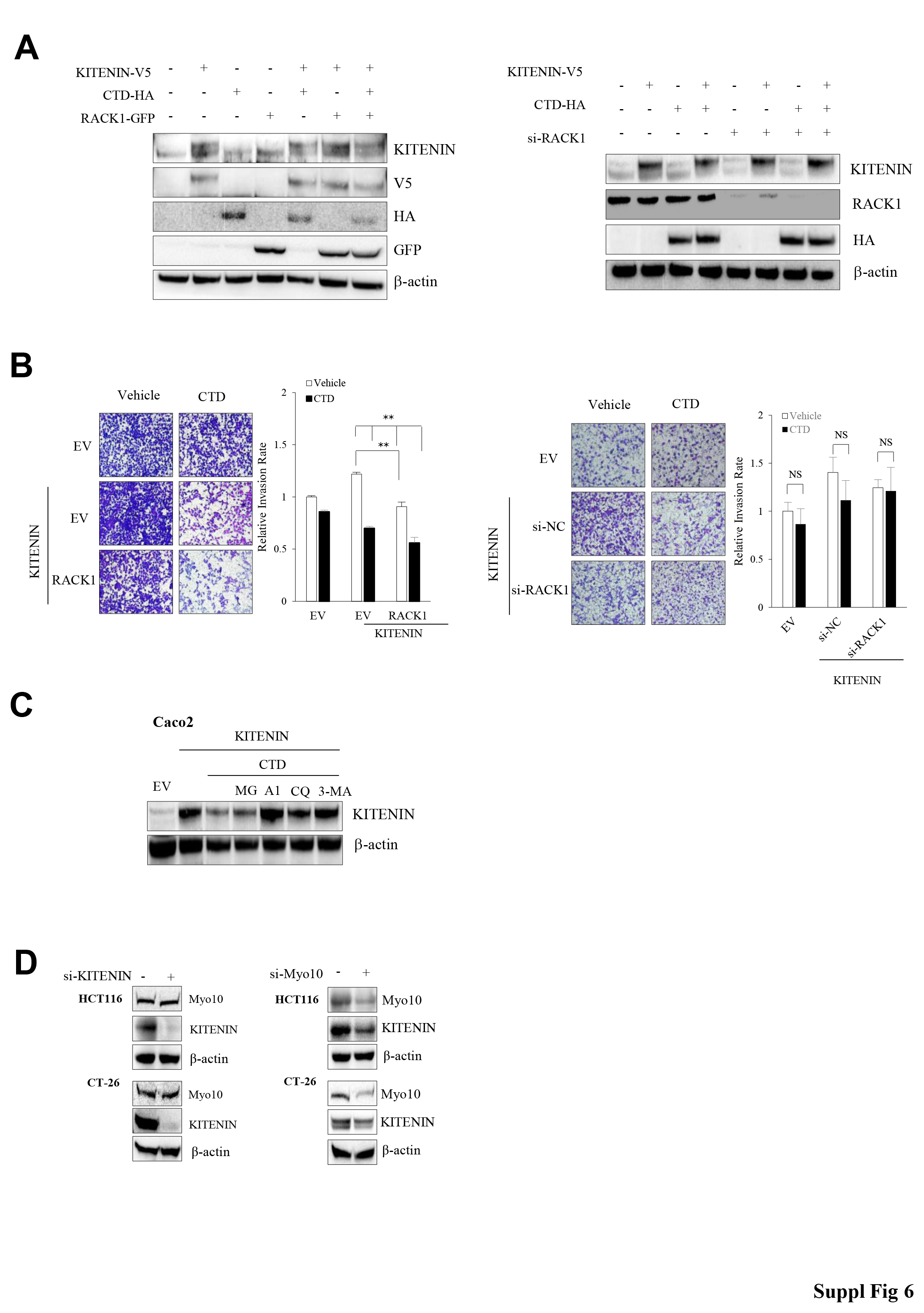

Supplement: Supplementary file 7 — Supporting FigureS6 Information [file CTM2-12-e871-s008.tif]

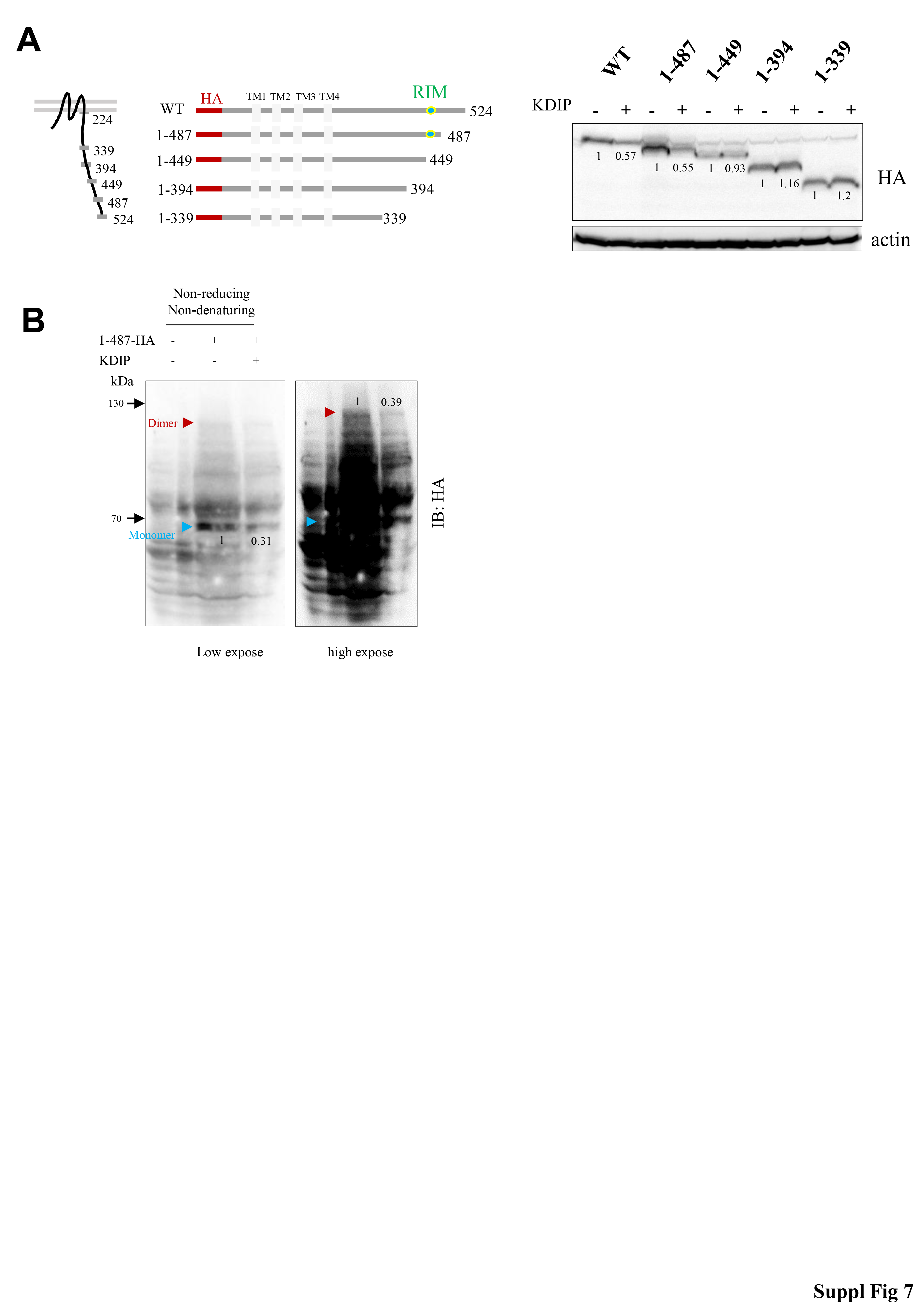

Supplement: Supplementary file 8 — Supporting FigureS7 Information [file CTM2-12-e871-s002.tif]

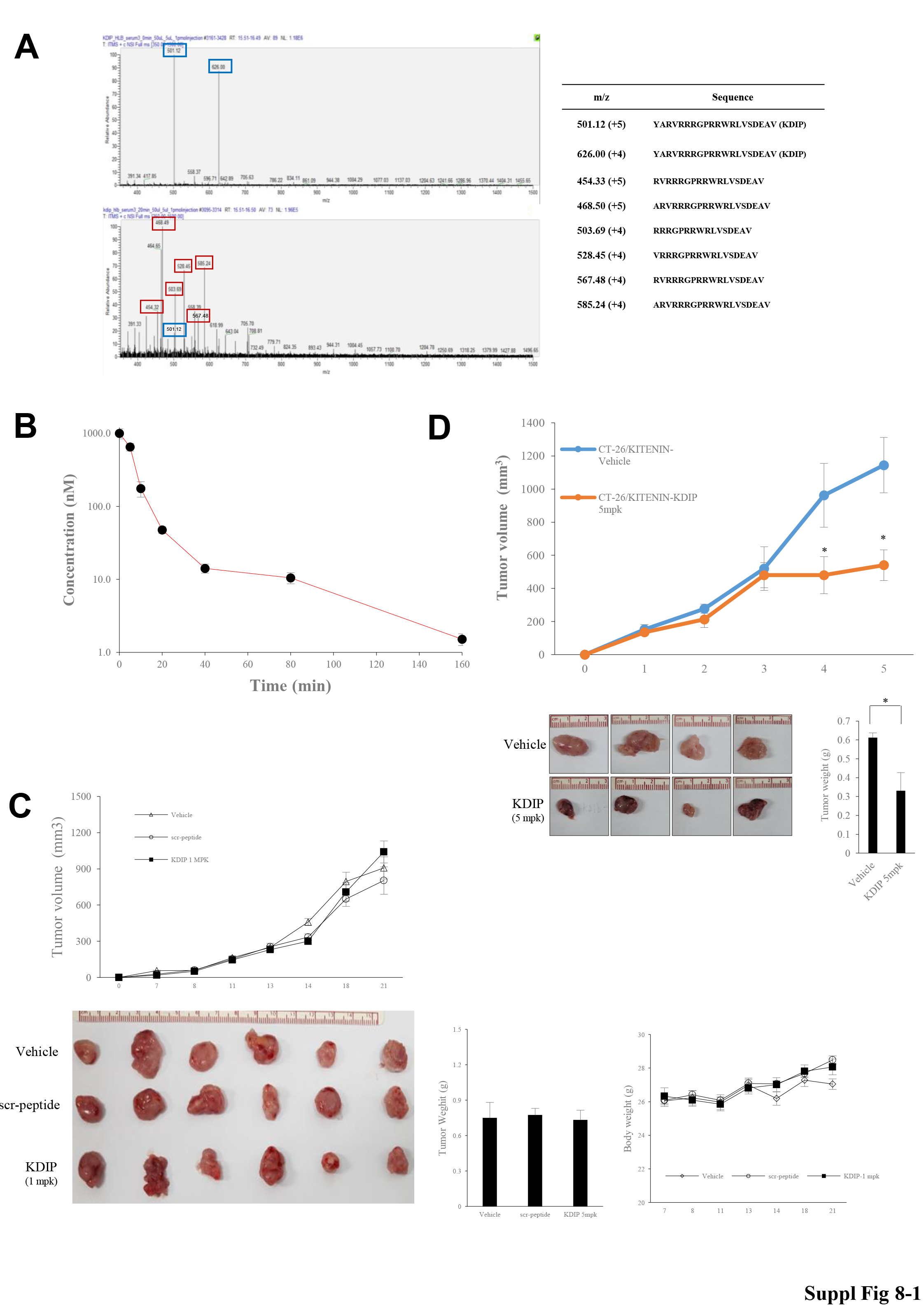

Supplement: Supplementary file 9 — Supporting FigureS8‐1 Information [file CTM2-12-e871-s005.tif]

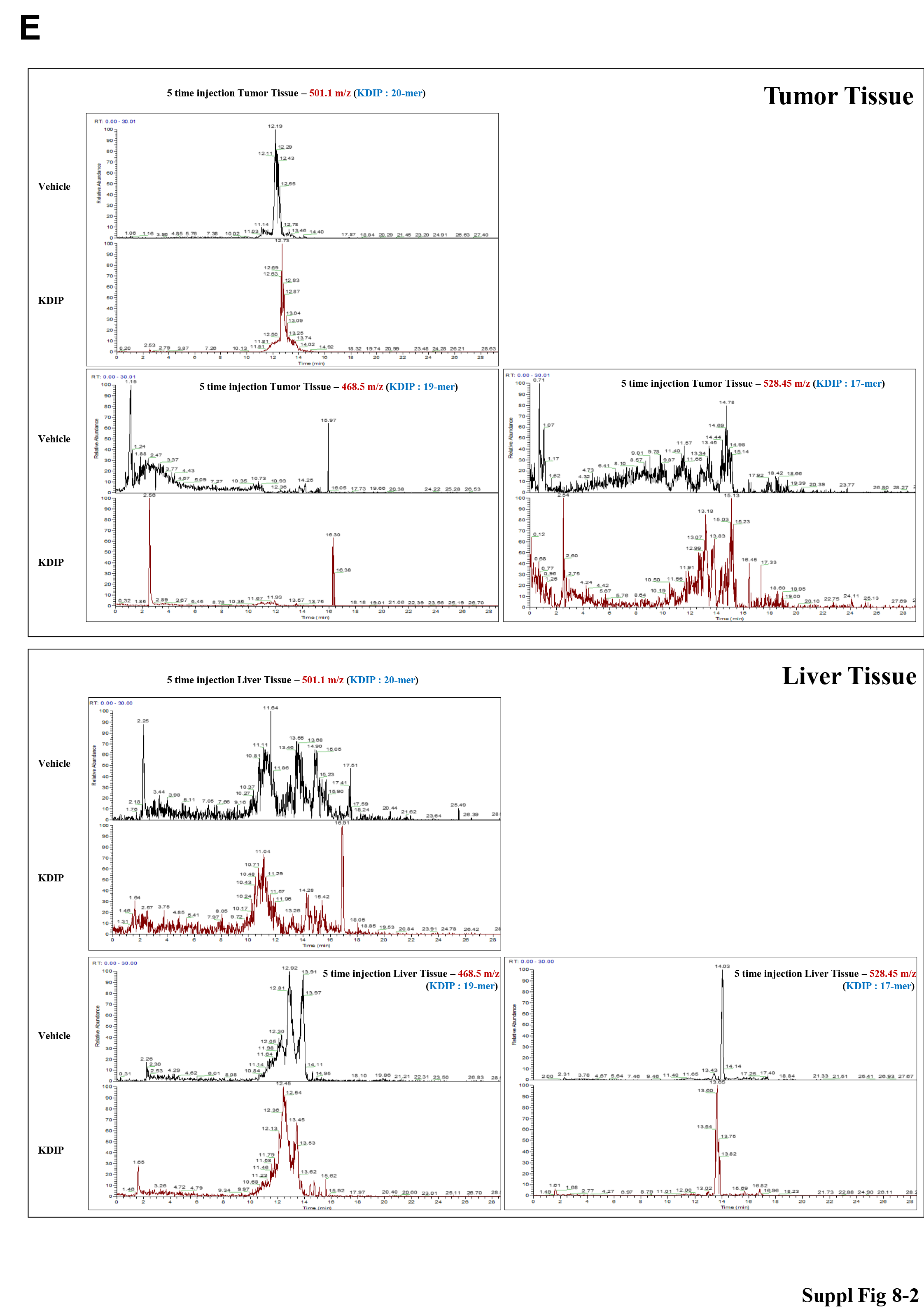

Supplement: Supplementary file 10 — Supporting FigureS8‐2 Information [file CTM2-12-e871-s007.tif]

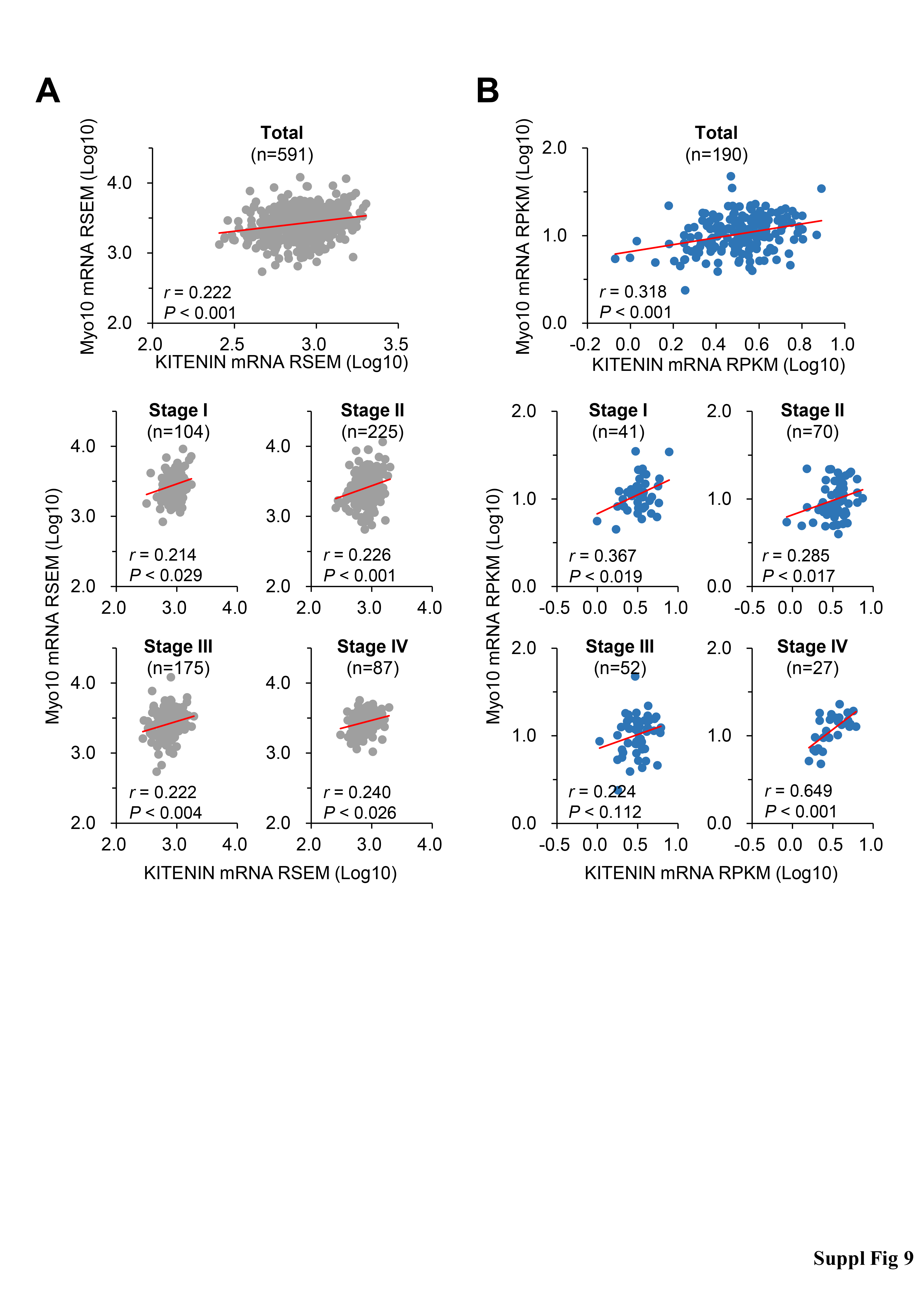

Supplement: Supplementary file 11 — Supporting FigureS9 Information [file CTM2-12-e871-s009.tif]
